# CYP1B1 is a potential target of kaempferol in renal fibrosis: a multi‑omics and single‑cell transcriptomics study

**DOI:** 10.1186/s40643-026-01135-z

**Published:** 2026-10-20

**Authors:** Chenjie Huang, Xueli Shi, Ziyou Yan

**Affiliations:** 1 School of Clinical Medicine, Jiangxi University of Chinese Medicine, Nanchang, Jiangxi Province China; 2 Zhuang Medicine College, Guangxi University of Chinese Medicine, Nanning, Guangxi Province China

**Keywords:** Kaempferol, Renal fibrosis, CYP1B1, Fibroblast activation, Single-cell transcriptomics, Virtual gene knockout

## Abstract

**Background:**

Renal fibrosis (RF) is a progressive and irreversible pathological process for which effective treatments are limited. This study explores the molecular targets and mechanisms through which kaempferol (Kae) ameliorates RF, with particular emphasis on cytochrome P450 1B1 (CYP1B1).

**Methods:**

Using a combination of transcriptomic profiling, weighted gene co‑expression network analysis (WGCNA), multi‑algorithm machine learning [least absolute shrinkage and selection operator (LASSO), support vector machine‑recursive feature elimination (SVM‑RFE), and Random Forest], and single‑cell transcriptomics, we identified the core targets of Kae intervention in RF. shapley additive explanations (SHAP) analysis evaluated gene contributions to the predictive model, while cell‑type identification by estimating relative subsets of RNA transcripts (CIBERSORT) estimated the immune microenvironment. The cell type-specific distribution of core targets was further characterized through single-cell RNA sequencing (scRNA-seq), and the scTenifoldKnk (a machine learning-based virtual gene knockout tool) algorithm simulated the effects of CYP1B1 loss-of-function on fibroblast activation and extracellular matrix (ECM) deposition. Finally, molecular docking was performed to predict the binding conformations of kaempferol with the proteins encoded by the six prioritized genes.

**Results:**

Six core genes (VCAM1, NFKBIA, SFN, CCL5, CYP1B1, and AXL) were identified from 25 Kae-RF-related genes, with CYP1B1 as an upstream regulatory hub. Immune infiltration analysis revealed increased plasma cells and eosinophils, alongside decreased M0/M1 macrophages and activated NK cells. Single-cell analysis localized CYP1B1 to smooth muscle cells and fibroblasts, with significant upregulation during late-stage fibroblast-to-myofibroblast differentiation. Virtual knockdown of CYP1B1 upregulated 46 genes associated with fibrosis-related biological processes, including cell adhesion, G protein-coupled receptor (GPCR) signaling, ion transport, and cytoskeletal remodeling. The six-gene diagnostic model achieved area under the curves (AUCs) of 0.889 (training) and 0.759 (external validation). Molecular docking showed favorable Kae–CYP1B1 binding (− 6.5 kcal/mol).

**Conclusion:**

CYP1B1 may serve as a potential functional target of Kae in RF, likely through modulation of fibroblast activation and ECM remodeling. These findings are hypothesis‑generating and provide a computational basis for future experimental validation of CYP1B1‑targeted strategies in RF.

**Graphical abstract:**

**Figure:**
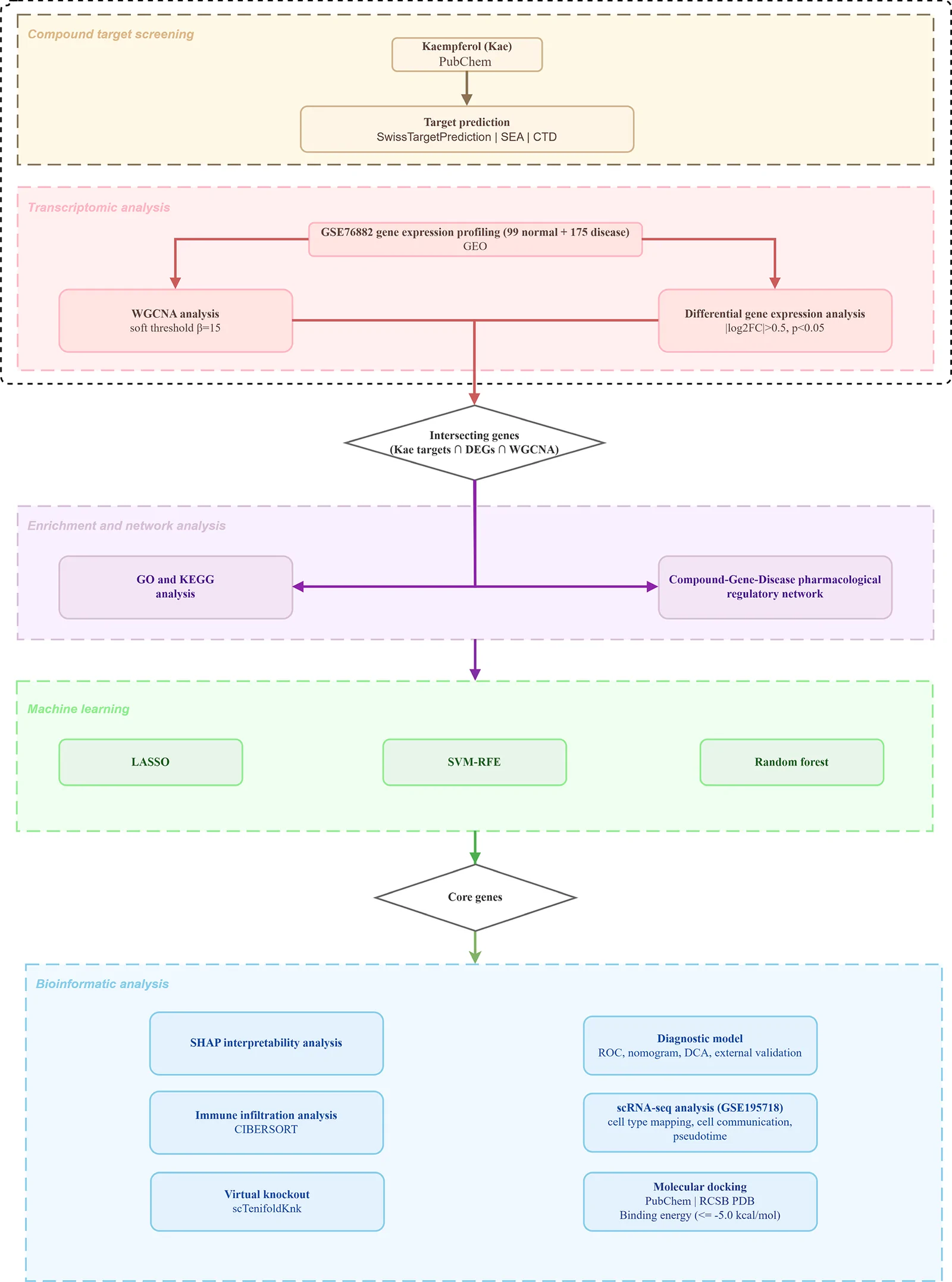

**Supplementary Information:**

The online version contains supplementary material available at https://doi.org/10.1186/s40643-026-01135-z.

## Introduction

Chronic kidney disease (CKD) has an insidious onset and a prolonged course, potentially progressing to end-stage renal disease without timely and effective interventions. This progression imposes significant health and economic burdens on both patients and society (Wang et al. 2016). Statistics indicate that CKD affects approximately 850 million individuals globally, representing about 9.1% of the global population. The prevalence continues to rise annually, with projections suggesting it will become the fifth leading cause of death worldwide by 2040 (Herrington et al. 2026). Renal fibrosis (RF) is a prevalent pathological feature associated with the progression of CKD to end-stage renal disease and serves as the fundamental pathological basis for irreversible renal tissue damage (Abbad et al. 2025; Ahui et al. 2026). Despite notable advancements in understanding the pathogenesis of RF in recent years, clinical treatment remains primarily focused on controlling risk factors and implementing comprehensive interventions, such as managing blood pressure, blood glucose, and proteinuria, as well as delaying the decline of renal function. However, specific antifibrotic drugs with targeted efficacy are still lacking (KDIGO 2024). Consequently, a thorough investigation into the pathological mechanisms of RF and the identification of new therapeutic targets hold substantial clinical significance. From a pathological perspective, RF is primarily characterized by tubulointerstitial fibrosis, glomerulosclerosis, and tubular atrophy. Its principal pathological feature is the abnormal deposition of extracellular matrix (ECM), which results in the destruction of renal tissue architecture and the progressive decline of renal function (Yuan et al. 2019; Zheng et al. 2025). This process is not attributable to a single factor; rather, it arises from a combination of pathological events, including persistent inflammatory responses, dysregulation of the immune microenvironment, injury to renal parenchymal cells, and abnormal activation of fibroblasts. These events involve a complex network of cytokines, inflammatory mediators, and signaling pathways (Liu 2024; Meng et al. 2025; Yamashita and Kramann 2024). Among these, the transforming growth factor-β (TGF-β)/sma and mad related family (Smad) pathway is the most extensively characterized mediator of myofibroblast differentiation and ECM deposition, while wingless (Wnt)/beta-catenin (β-catenin) signaling has also been implicated in fibroblast activation and epithelial–mesenchymal transition (Abbad et al. 2025; Liu 2024). Oxidative stress and chronic inflammation are key drivers of fibrotic progression, mediated in part through nuclear factor kappa-B (NF-κB) and nuclear factor erythroid 2-related factor 2 (Nrf2) pathways, forming a vicious cycle of cellular injury and fibrotic remodeling (Liu et al. 2025a, b, c). Emerging evidence also highlights the aryl hydrocarbon receptor (AhR) pathway as a critical mediator of tubulointerstitial fibrosis, with AhR activation promoting myofibroblast differentiation and ECM deposition (Cao et al. 2022). In addition, metabolic reprogramming—including dysregulated phospholipid metabolism—and gut microbiota dysbiosis have been identified as contributing factors to RF pathogenesis (Liu et al. 2025a, b, c; Wang et al. 2023a, b; Yao et al. 2026). Recent studies indicate that the pathological transdifferentiation of fibroblasts into myofibroblasts is a crucial factor driving the progression of RF (Lathan 2024). Under continuous stimulation from multiple pro-fibrotic signals, fibroblasts undergo transcriptional and phenotypic reprogramming, which is characterized by increased ECM synthesis and resistance to apoptosis. This ultimately leads to the deposition of fibrotic scars and a continuous deterioration of renal function (Liao et al. 2024; Yamashita and Kramann 2024). However, the key molecular nodes and their interaction networks that regulate these processes remain inadequately characterized. Consequently, identifying molecular targets that can effectively intervene in critical pathological processes—such as the abnormal activation of mesenchymal cells, the remodeling of the immune microenvironment, and the persistent inflammatory response—along with investigating their mechanisms of action, has emerged as a significant research focus in the domain of RF prevention and treatment.

Cytochrome P450 1B1 (CYP1B1) is a crucial metabolic enzyme within the cytochrome P450 superfamily, responsible for the metabolism of a diverse array of endogenous and exogenous substrates. Additionally, it has been reported to play a significant role in the development of multiorgan fibrosis by regulating oxidative stress, arachidonic acid metabolism, and inflammatory cascade responses (Kim et al. 2025; Wang et al. 2019). Fibroblasts are the primary source of myofibroblasts inRF, making them a critical cell type for understanding fibroblast activation and ECM deposition. Although preliminary evidence has indicated the involvement of CYP1B1 in RF (Zhang et al. 2025a, b, c), its expression dynamics, cellular origins, and functional localization in RF remain inadequately characterized, particularly due to the absence of studies focusing on specific cellular subpopulations at single-cell resolution. Given that RF represents a complex pathological process influenced by the collaborative actions of multiple cell types, investigating the dysregulated expression of CYP1B1 across various renal cell lineages and its correlation with fibrosis progression may yield valuable insights for targeted therapeutic interventions. Given its upstream regulatory position, identifying natural compounds capable of modulating CYP1B1 represents a promising strategy. Natural products, recognized for their multi-targeting capabilities and low toxicity, have emerged as significant sources for the development of anti-RF drugs (Zhang et al. 2025a, b, c). Consequently, identifying natural active compounds that can modulate CYP1B1 for the prevention and treatment of RF holds substantial research potential.

In recent years, traditional Chinese medicine (TCM) has attracted increasing attention as a promising source of therapeutic agents for RF, owing to its multi‑component, multi‑target characteristics and long‑standing clinical experience. Extensive research has indicated that TCM exhibits unique advantages and promising efficacy in the treatment of RF (Acquah-Mills et al. 2025; Cai et al. 2025; Li and Li 2025). TCM formulations, such as Yiqi Wenyang Formula, Niaoduqing particles, Yishenqinglihuoxue formula, and Uremic Clearance Granules, have been reported to ameliorate RF and delay CKD progression through multiple mechanisms, including amelioration of gut microbiota dysbiosis, modulation of bile acid and tryptophan metabolism, and suppression of inflammatory pathways such as IκB/NF-κB signaling (Liu et al. 2026a, b, c; Wu et al. 2025; Yuan et al. 2025). Furthermore, single herb extracts, such as those from Rhubarb, Tripterygium wilfordii, Astragalus membranaceus, and Panax notoginseng, have also shown protective effects against kidney injury and fibrosis through anti-inflammatory, antioxidant, and immunomodulatory activities (Gao et al. 2025; Miao et al. 2025, 2022). Moreover, a growing body of evidence indicates that natural compounds derived from TCM sources—including naringin, hederagenin, morin, geniposidic acid, apigenin, and acteoside—exert renoprotective effects by modulating oxidative stress, inflammation, and key signaling pathways such as Janus kinase/signal transduction and transcription activation (JAK/STAT), Nrf2, and AhR signaling (Ajibade et al. 2024; Ben-Azu et al. 2025; Miao and Vaziri 2025; Wang et al. 2024). Among these, kaempferol (Kae), a natural flavonoid, exhibits a range of pharmacological activities, including anti-inflammatory, antioxidant, antitumor, and antifibrotic effects (Imran et al. 2019; Liu et al. 2025a, b, c). Kae has been shown to alleviate fibrosis in various organs, including the liver, heart, lungs, and kidneys, by modulating oxidative stress, inflammatory cell infiltration, collagen deposition, and fibrogenic factors (Cao et al. 2026; Shi et al. 2025; Zhang et al. 2025a, b, c). Despite the promising therapeutic effects of Kae on multiple organ fibrosis, its molecular mechanism of action in RF remains unclear. Most existing studies have concentrated on tissue-level responses or specific epithelial cells, without systematically examining whether Kae can directly intervene in the activation of renal mesenchymal cells, the pathological transdifferentiation of fibroblasts, or the transcriptional reprogramming associated with fibrosis. Furthermore, given the multi‑target nature of naturally active small molecules and the limitations of traditional single‑target approaches, systematic studies at the single‑cell level are needed to computationally explore whether Kae may exert anti‑RF effects through CYP1B1‑associated pathways, thereby generating testable hypotheses and constructing related cellular regulatory networks. Moreover, conventional single-target approaches are insufficient to capture the synergistic actions of kaempferol within the fibrotic milieu, largely because evidence is scarce concerning how lineage-specific regulatory networks operate at cellular resolution. Consequently, single-cell transcriptomics, in combination with other advanced platforms, offers a promising strategy to decipher how CYP1B1 exerts cell-lineage-dependent pathological effects during RF progression. Such analyses may further clarify the potential interventional effects of Kae on relevant cellular subpopulations and their regulatory networks. The NF-κB signaling pathway serves as a crucial nexus connecting inflammatory activation and fibrotic remodeling (Yuan et al. 2022), and Kae has been shown to inhibit NF-κB activity across various disease models (Alshehri et al. 2022; Herrera et al. 2025). Given this background and the inflammatory signatures identified in our preliminary enrichment analysis, we sought to computationally prioritize whether CYP1B1 is more closely transcriptionally linked to mesenchymal activation (ECM remodeling) rather than purely inflammatory pathways, based on subsequent virtual perturbation and single‑cell trajectory outcomes.

**Fig. 1:**
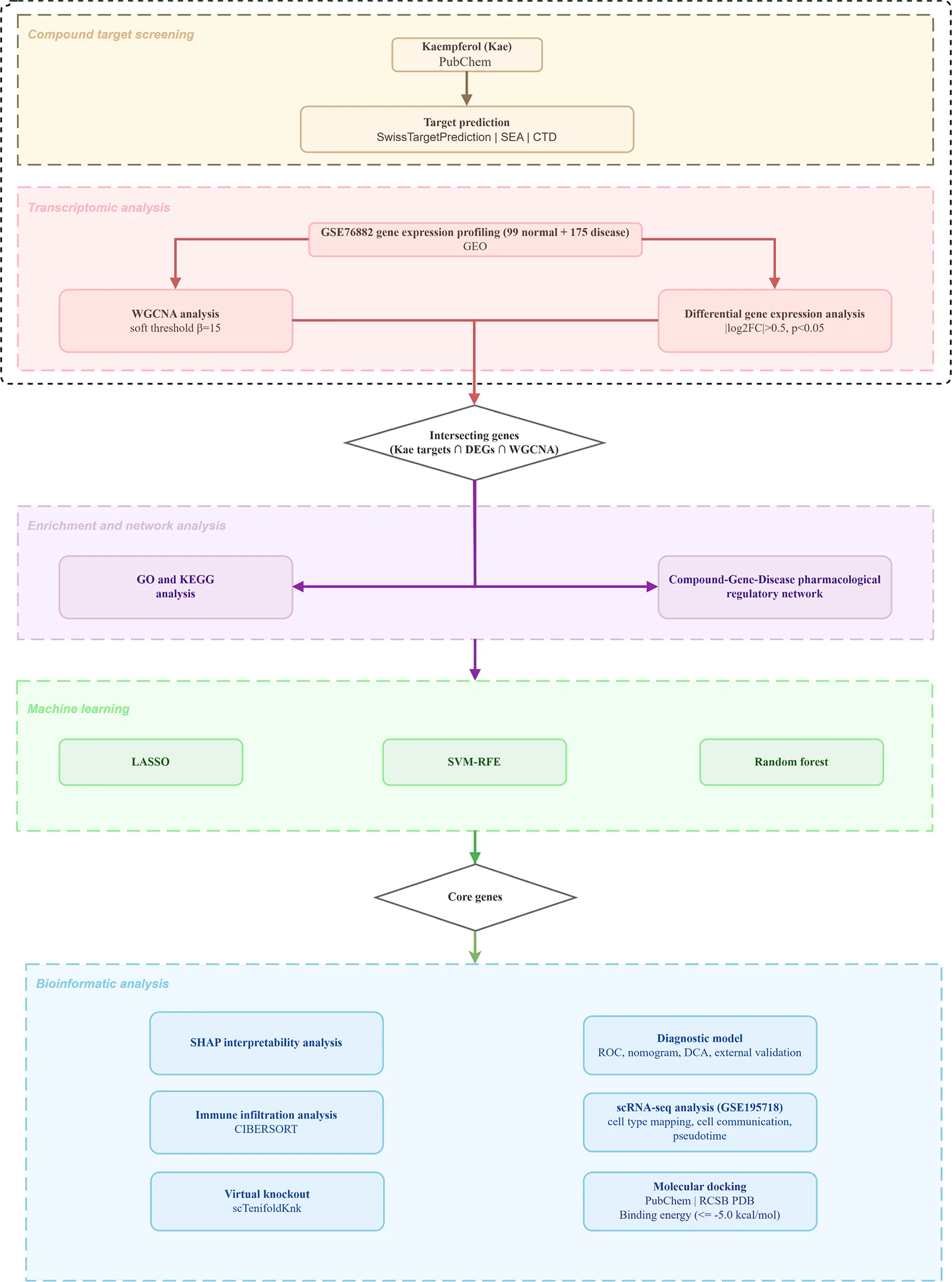
Schematic workflow of the study. The chart delineates the multi-stage process encompassing target prediction, transcriptomic profiling, machine learning, diagnostic modeling, single-cell analysis, virtual knockout, and molecular docking to computationally predict candidate targets and regulatory networks of Kae in RF

Network pharmacology combined with molecular docking is recognized as a powerful computational strategy for systematically exploring the therapeutic mechanisms of natural products and identifying potential drug targets (Shahzadi et al. 2024). Addressing the identified research gaps, this study employed transcriptome differential analysis, weighted gene co-expression network analysis (WGCNA), various machine learning algorithms, and single-cell transcriptomics techniques to construct a core target mapping of Kae intervention in RF from the bulk to the single-cell level. The focus was on predicting the cell-specific role of CYP1B1 and its functional regulatory network. We proposed that CYP1B1 may serve as a critical regulatory node for fibroblast activation and phenotypic remodeling in RF, extending beyond its involvement in inflammatory signaling. To computationally evaluate this proposed role, we performed pseudotime analysis to reveal the expression dynamics of CYP1B1 during the differentiation of fibroblasts into myofibroblasts. Additionally, we employed scTenifoldKnk (a machine learning-based virtual gene knockout tool) to simulate the perturbative effects of CYP1B1 loss of function on the downstream transcriptional network, thereby inferring its predominant biological function. Furthermore, we assessed the binding affinity of Kae to CYP1B1 through molecular docking and integrated the functional characteristics of CYP1B1 with the established anti-inflammatory and anti-fibrotic pharmacological profiles of Kae, as well as insights related to the NF-κB pathway. This comprehensive approach aims to provide a theoretical foundation and identify potential targets for precise intervention in RF. Given the purely computational and hypothesis‑generating nature of this study, all findings are intended to prioritize candidate targets for future experimental validation rather than to establish definitive causal relationships. The overall analytical workflow of this study is illustrated in Fig. 1.

## Methods and materials

### Kae target prediction and RF transcriptome data organization

The chemical structure of kaempferol (PubChem CID: 5,280,863) was retrieved from the PubChem database (https://pubchem.ncbi.nlm.nih.gov/, accessed on 19 March 2026). To predict its potential targets, three online databases—SwissTargetPrediction (http://www.swisstargetprediction.ch/, accessed on 19 March 2026), SEA (https://sea.bkslab.org/, accessed on 19 March 2026), and CTD (https://ctdbase.org/, accessed on 19 March 2026)—were utilized, focusing on the species Homo sapiens. Candidate genes from SwissTargetPrediction were selected based on a minimum probability score of 0.05, while SEA and CTD include all human target genes. The predicted targets from these three databases were subsequently merged, de-duplicated, and normalized using the UniProt database (https://www.uniprot.org/, accessed on 19 March 2026) to establish the final target gene set for Kae. The transcriptome dataset GSE76882 (platform: GPL13158) of RF was downloaded from the NCBI GEO (https://www.ncbi.nlm.nih.gov/geo/, accessed on 19 March 2026) database, which comprises 99 normal samples and 175 disease group samples. For differential gene screening, we applied the limma algorithm (v3.66.0) within the R computational framework (v4.5.1; R Foundation for Statistical Computing, Vienna, Austria). Prior to analysis, the original expression matrix was normalized by Z-score to eliminate scale differences among various gene expression levels. Subsequently, principal component analysis (PCA) was employed to reduce the dimensionality of the overall expression profile characteristics of the samples, revealing a discernible trend of separation between the normal and disease groups in the principal component space. Using |log_2_FC|> 0.5 and *P* < 0.05 as the criteria for identifying differentially expressed genes (DEGs), the samples were further analyzed employing the “pheatmap (version 1.0.13)” and “ggplot2 (version 4.0.2)” packages in R. Using these tools, we generated heatmaps to reveal sample-level clustering of DEGs and volcano plots to depict the relationship between expression fold-change and statistical significance.

### WGCNA module identification and network topology screening

In this study, the “WGCNA (version 1.74)” package in R was employed to construct a weighted gene co-expression network. Initially, samples were clustered using the hierarchical clustering method, with a cutHeight set to 20,000 to identify and exclude outlier samples. Subsequently, a soft‑threshold power of β = 15 was selected to ensure a scale‑free topology fit index (R^2^) above 0.9. To identify co‑expression modules, we applied the dynamic tree‑cutting procedure (implemented via the cutreeDynamic function), using a minimum module size of 50 and a merging height cutoff of 0.25. Visualization of the gene dendrogram and module assignments was accomplished with the plotDendroAndColors function. For each module, we extracted the first principal component as its module eigengene (ME). Pearson correlation analysis was then performed between each ME and the phenotypes of interest, and modules with |R|> 0.5 and *P* < 0.05 were retained as significantly correlated. The correlation results were summarized as a module‑trait association heatmap. Candidate core genes within each module were then defined by requiring both module membership (kME > 0.8) and gene significance (GS > 0.2). The genes showing the strongest phenotype association—defined by the lowest *P*‑value and |R|> 0.5—were ultimately prioritized as candidates for downstream functional investigation. In addition, we performed parameter sensitivity analyses near the optimal soft threshold to assess the robustness of WGCNA parameter selection.

To identify key genes associated with disease and drug action, we intersected the core module genes from WGCNA with DEGs, defining this intersection as the disease-related gene set for RF. Subsequently, we further intersected this set with the target genes of Kae, resulting in the Kae-RF-related gene set. Venn diagrams were generated using the ggvenn package (v0.1.19) to display the overlapping results. The network relationship files and node attribute files were subsequently prepared and fed into Cytoscape (v3.10.3; Cytoscape Consortium, La Jolla, CA, USA). Within Cytoscape, the overlapping genes were categorized and visualized based on three node attributes: “Disease (RF)”, “Compound (Kae)”, and “Gene (intersecting target gene)”. Through this classification and mapping, we constructed the Kae‑RF association network to illustrate the predicted interactions among compounds, diseases, and their intersecting targets.

### Pathway and functional annotation of the candidate gene set

To gain insight into the potential biological processes modulated by Kae in RF, the clusterProfiler package (v4.18.4) was applied, using Org.Hs.eg.db as the reference annotation, to perform gene ontology (GO) term and kyoto encyclopedia of genes and genomes (KEGG) pathway overrepresentation analyses on the 25 Kae‑RF overlap genes. This analysis aimed to annotate the overall functional background of the Kae-RF target genes and to provide directional insights for subsequent studies, with *P* < 0.05 set as the significance threshold. For the intersecting targets, we performed GO annotation covering the three standard ontologies: BP (biological process), CC (cellular component), and MF (molecular function) (Yu et al. 2012). This enabled the classification of target genes according to their biological roles, subcellular distribution, and molecular activities. To explore the molecular basis of Kae’s action against RF, we carried out KEGG enrichment analyses to pinpoint pathways with a significant accumulation of target genes. This approach allowed us to infer the candidate signaling routes and metabolic processes potentially involved in Kae‑mediated renoprotection. Visualization of the enriched functional classes and signaling pathways was facilitated through the “enrichplot (version 1.30.5)” package.

### Machine learning framework construction and SHAP interpretability analysis

To identify the core genes of Kae that regulate RF, three machine learning algorithms—least absolute shrinkage and selection operator (LASSO) regression, support vector machine‑recursive feature elimination (SVM‑RFE), and Random Forest—were integrated in this study. The LASSO regression utilized the R package “glmnet (version 4.1.10)”, with parameters configured as alpha=1 and family=binomial. Using 10‑fold cross‑validation (nfolds=10), we identified λ as the optimal penalty parameter, leading to the retention of non‑zero coefficients as candidate feature genes. SVM-RFE analysis was conducted using the “e1071 (version 1.7.17)” package in R. To further refine the key features identified through LASSO dimensionality reduction, this study used the candidate genes from LASSO as input variables. A linear kernel support vector machine model was constructed after standardizing the gene expression data, with the cost parameter set to cost=10, and the model’s performance was evaluated using 10-fold cross-validation. During recursive feature elimination, half of the features were removed per round once the count surpassed 50, with their importance scores subsequently evaluated based on average rankings. The Random Forest model was developed using the “randomForest (version 4.7.1.2)” package in R. The Kae‑RF‑related genes served as input variables, with the number of decision trees initially set to ntree=500, and the optimal number of trees was determined by monitoring changes in the out-of-bag (OOB) error rate (> 50). The importance of genes was assessed using the MeanDecreaseGini index, leading to the ranking of the top 10 genes in descending order of significance as candidate genes for further characterization. The genes identified through the screening of the three algorithms were considered the intersection of the core candidate genes. To explore the potential of these core genes as diagnostic indicators for RF, we generated box plots with the ggplot2 package (v4.0.2). These plots illustrated the expression distributions of each candidate gene between the control and disease cohorts, using the transcriptome‑wide differential expression profiles. To evaluate the diagnostic performance of each prioritized gene, we used its normalized expression value as the predictor and classified the RF patients versus normal controls as the binary outcome, with receiver operating characteristic (ROC) curves generated via the pROC package (v1.19.0.1). The area under the curve (AUC) was utilized to assess the diagnostic efficacy of each core gene, with an AUC greater than 0.70 indicating superior diagnostic value.

Based on single-gene diagnostic analysis, this study developed a multigene joint diagnostic model. Multifactorial logistic regression analysis was employed to evaluate the independent risk effects of the core genes, and the odds ratio (OR) along with 95% confidence intervals (CI) for each gene were calculated and visualized using forest plots. Additionally, a nomogram was created based on the regression coefficients to integrate multiple core genes and predict individual disease risk. The model’s fit was assessed through a calibration curve, while the net clinical benefit was evaluated using decision curve analysis (DCA). These analyses utilized the “rms (version 8.1.1)”, “regplot (version 1.1)”, “rmda (version 1.6)”, “pROC (version 1.19.0.1)”, and “ggplot2 (version 4.0.2)” packages. Furthermore, an independent external validation set, GSE22459 (platform: GPL570) (comprising 25 samples from the normal group and 40 samples from the disease group), was employed to validate the diagnostic performance and generalizability of the model. To determine the overall diagnostic power of the combined model, we constructed ROC curves using both the training and validation datasets.

To gain deeper insight into the model’s decision-making process, we applied shapley additive explanations (SHAP) to quantify each feature’s contribution to the predictions of our conventional machine learning models. We calculated SHAP values with the kernelshap package (v0.9.1) to measure each feature’s contribution to the prediction outcome. The resulting values were then displayed as importance histograms, summary plots, dependency scatter plots, and single‑sample force plots via shapviz (v0.10.3) and ggplot2 (v4.0.2), collectively revealing the direction and strength of each feature’s effect.

### Immune cell infiltration and core gene‑immune correlation analysis

We used the cell‑type identification by estimating relative subsets of RNA transcripts (CIBERSORT) algorithm to evaluate immune cell infiltration in the RF transcriptomic data, estimating the relative abundance of 22 immune cell subpopulations in each sample. Differences in immune cell composition between the two sample groups were visualized using box-and-line plots, while between-group comparisons were conducted with the Wilcoxon test [*p*-values corrected for false discovery rate (FDR)]. The relationship between core gene expression levels and immune cell infiltration abundance was further evaluated through Spearman correlation analysis. We further examined the correlations between each prioritized gene and the infiltrating immune cells. These associations were visualized as heatmaps using the linkET package (v0.1.0). Additionally, the correlation network was constructed with the same package, where the thickness and color of the lines denoted the strength and direction of the correlations. Significance of the correlations was indicated by *P* < 0.05, based on FDR correction.

### Single-cell transcriptome mapping and functional analysis

Single-cell RNA sequencing data were sourced from the GEO database (GSE195718, platform: GPL24676), encompassing a total of 9 samples. Following quality control, cells with fewer than 300 genes or greater than 20% mitochondrial gene content were filtered out. Doublets were identified and excluded using the “scDblFinder (version 1.24.10)” package in R. For single-cell transcriptomic profiling, we used the Seurat toolkit (v5.4.0) and initialized the random seed with 666. Violin plots illustrate the number of genes and sequencing depth for each sample prior to filtering, along with the total number of cells retained after filtering. The “clustree (version 0.5.1)” package was employed to evaluate the stability of clustering results across a resolution range of 0.1–1.0 and to ascertain the optimal clustering parameters. Cell clustering was executed based on the first 2500 highly variable genes using 15 principal components (PCs) at a resolution of 0.3. The “SingleR (version 2.12.0)” package was then utilized for cell annotation, referencing the Human Primary Cell Atlas and incorporating manual calibration. Finally, the distribution of cell subpopulations was visualized using Uniform Manifold Approximation and Projection (UMAP) for dimensionality reduction. The FeaturePlot and VlnPlot functions were employed to illustrate the expression distribution of core genes in UMAP space and to depict their expression levels across various cell types, respectively. Intercellular communication analysis was conducted using the “CellChat (version 2.2.0.9001)” package, utilizing the human ligand-receptor database (CellChatDB.human) to compute communication probabilities among cell subpopulations while filtering low-confidence interactions (min.cells= 10). Ligand-receptor pairs were inferred through the computeCommunProb function (raw.use=TRUE), and the communication network among cell types was visualized by aggregating the network via aggregateNet and generating an overall network graph that represents the number and strength of interactions using the netVisual_circle function. Furthermore, the “monocle3 (version 1.3.7)” package in R was utilized to construct cell developmental trajectories, using fibroblasts as the developmental starting point to trace the dynamics of cell differentiation. Finally, the “scTenifoldKnk (version 1.0.3)” package was employed to simulate the virtual knockdown effect of core genes, with parameters set to qc_mtThreshold= 0.2, qc_minLSize= 1000, nc_nNet= 3, and nc_nCells= 300, in conjunction with a non-targeting control to account for potential background perturbations. This analysis aimed to assess the transcriptional perturbations resulting from CYP1B1 knockdown on the identified cell lineages, identify significantly differentially expressed genes, and systematically summarize the biological processes associated with these effects in conjunction with existing literature data.

### Molecular docking validation

We performed molecular docking to evaluate the binding potential of kaempferol against the core protein targets. The ligand coordinates were sourced from PubChem (https://pubchem.ncbi.nlm.nih.gov/, accessed on 19 March 2026), while receptor coordinates for the six proteins (VCAM1: PDB ID [1VSC]; CYP1B1: PDB ID [3PMO]; CCL5: PDB ID [5CMD]; SFN: PDB ID [1YWT]; NFKBIA: PDB ID [9T9W]; AXL: PDB ID [5U6B]) were acquired from the RCSB PDB (https://www.rcsb.org/, accessed on 5 April 2026). The receptor structures were prepared in PyMol (v3.1.6.1, Schrödinger, LLC, New York, NY, USA) by stripping water and proto‑ligands, excluding non‑functional cofactors, and adding polar hydrogens to the protein models. Prior to docking, we defined the active-site center of each receptor using AutoDockTools (v1.5.7, The Scripps Research Institute, La Jolla, CA, USA) and adjusted the grid box dimensions based on the ligand size. Automated docking was carried out with AutoDock Vina (v1.1.2, The Scripps Research Institute, La Jolla, CA, USA), and an energy threshold of ≤ − 5.0 kcal/mol was used as the cutoff to preselect candidate binding poses. Finally, the conformations exhibiting optimal binding energies were visualized using PyMol 3.1.6.1 software to analyze the ligand molecules in relation to their binding sites, hydrogen bonds, and other key interaction types on the target proteins.

## Results

### Target prediction and DEG screening

**Fig. 2:**
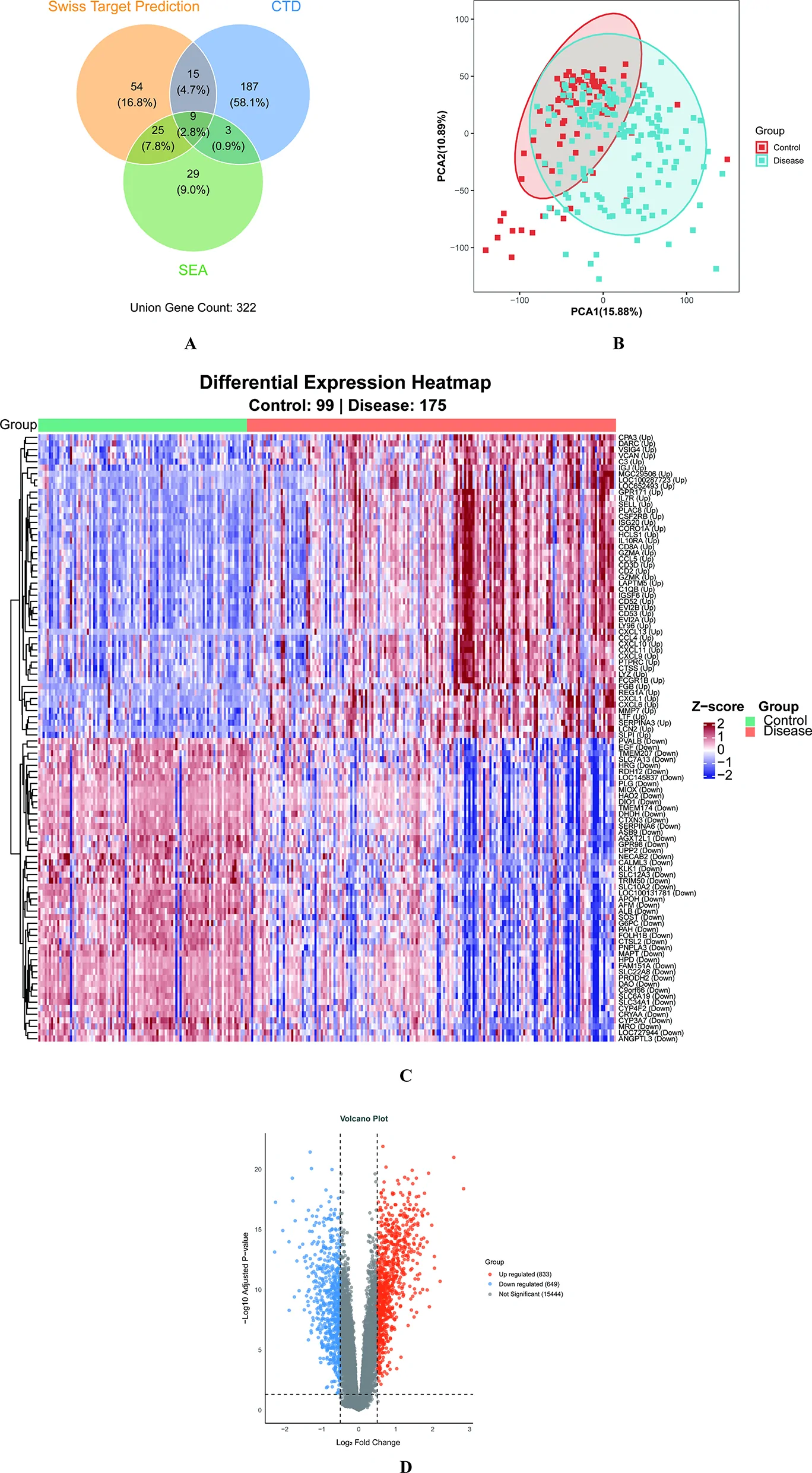
kaempferol (Kae) target prediction and renal fibrosis (RF) transcriptome difference analysis. **A** Venn diagram showing the overlap of predicted Kae targets among SwissTargetPrediction, CTD, and SEA (n=322). **B** principal component analysis (PCA) plot indicating a trend of separation between disease and control samples. **C** Heatmap displaying the top 50 up‑ and down‑regulated differentially expressed genes (DEGs) across 99 control and 175 disease samples. Expression values are Z‑score‑normalized (red: high; blue: low), with hierarchical clustering applied to both genes and samples. DEGs were identified using the limma package with thresholds of |log_2_FC|> 0.5 and *P* < 0.05. **D** Volcano plot of 1,482 DEGs, with log_2_FC (disease vs. control) on the X‑axis and −log₁₀(*P*‑value) on the Y‑axis. Red: up‑regulated (log_2_FC > 0.5, *P* < 0.05); blue: down‑regulated (log_2_FC < −0.5, *P* < 0.05); grey: not significant (|log_2_FC|≤ 0.5 or *P* ≥ 0.05). Red and blue point separation illustrates the opposing directions of expression changes. The horizontal dashed line indicates *P* = 0.05, and the vertical dashed lines indicate log_2_FC = ±0.5

In this study, three online databases—SwissTargetPrediction, SEA, and CTD—were integrated for target prediction, resulting in the identification of 322 potential target genes for Kae after merging, deduplicating, and normalizing the prediction results (Fig. 2A). Concurrently, the transcriptome dataset GSE76882 of RF, which includes 99 control samples and 175 disease group samples, was downloaded from the GEO database. PCA revealed a discernible trend of separation between the two sample groups in the principal component space (Fig. 2B). Using the limma package (v3.66.0) in R, we applied thresholds of |log2FC|> 0.5 and a *P*‑value < 0.05 to screen for DEGs. This yielded 1,482 DEGs in total, consisting of 833 up‑regulated and 649 down‑regulated genes. The differences in the distribution and expression patterns of these DEGs were visualized through a volcano plot and clustered heatmap (Fig. 2C–D).

### WGCNA module identification and association network construction

**Fig. 3:**
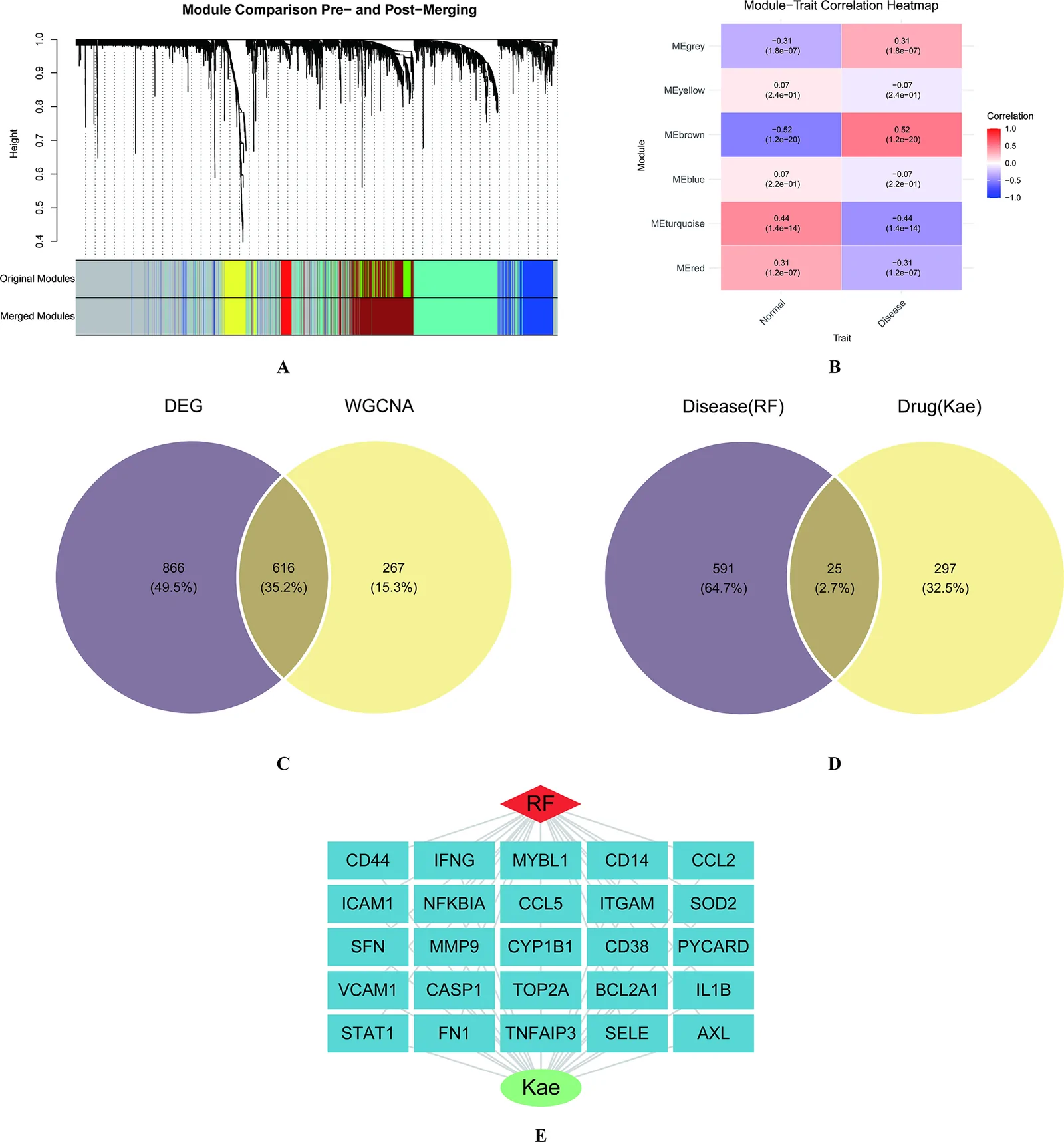
Kae-RF-related gene screening and association network construction. **A** Hierarchical clustering tree of co‑expressed genes with module color assignments below, revealing six distinct co‑expression modules identified using WGCNA (soft threshold β = 15, scale‑free topology R^2^ > 0.9). **B** Module‑trait association heatmap showing correlations between each module and disease status, with each cell annotated with correlation coefficients and *P*‑values. Module eigengenes (MEs) were extracted as the first principal component of each module. The brown module shows the strongest positive correlation with the RF phenotype (|R|= 0.52, *P* < 0.05). **C** Overlap between the brown module genes (n = 883) and DEGs (n = 1,482), yielding 616 disease‑associated genes. **D** Another Venn diagram depicts the intersection of disease-associated genes for RF (n=616) with drug targets for Kae (n=322), yielding a total of 25 Kae-RF-associated genes. **E** The Kae-RF association network diagram presents the pharmacological regulatory network involving compounds, genes, and diseases. Green, blue, and red nodes represent compounds, genes, and diseases, respectively, while the connecting lines indicate predicted interactions

In this study, WGCNA was employed to identify gene co-expression modules closely associated with RF. A soft threshold power of 15 was established based on the scale-free topology criterion (R^2^ > 0.9), resulting in the identification of six co-expression modules using the dynamic tree-cutting method (Fig. 3A). Module-trait association analysis indicated that the brown module, comprising 883 genes, exhibited the most significant correlation with disease status (|R|= 0.52, *P* < 0.05) and was designated as the core module (Fig. 3B). Subsequently, the genes from the core module were intersected with DEGs, yielding a total of 616 genes (Fig. 3C), which were classified as the disease gene set. These genes were then intersected with the predicted targets of Kae (the drug gene set), resulting in the identification of 25 Kae-RF-related genes (Fig. 3D and E). To evaluate the robustness of the WGCNA module identification and core gene selection, we conducted parameter sensitivity analyses near the optimal soft threshold (β = 15) (13–17) and at varying combination heights (0.20–0.30).The results indicated that the significant modules predominantly consisted of brown or blue modules across various parameter combinations. The intersections of the module genes with DEGs and Kae targets were sequentially analyzed for each parameter. At the optimal soft threshold (β=15), 25 Kae-RF-related genes were accurately identified, a number that closely aligns with the intersections observed under other parameters (20–25) (Supplementary Material S1). This finding suggests that the candidate gene set identified in this study is robust with respect to the selection of WGCNA parameters.

### Functional annotation and pathway enrichment of the Kae‑RF intersecting genes

**Fig. 4:**
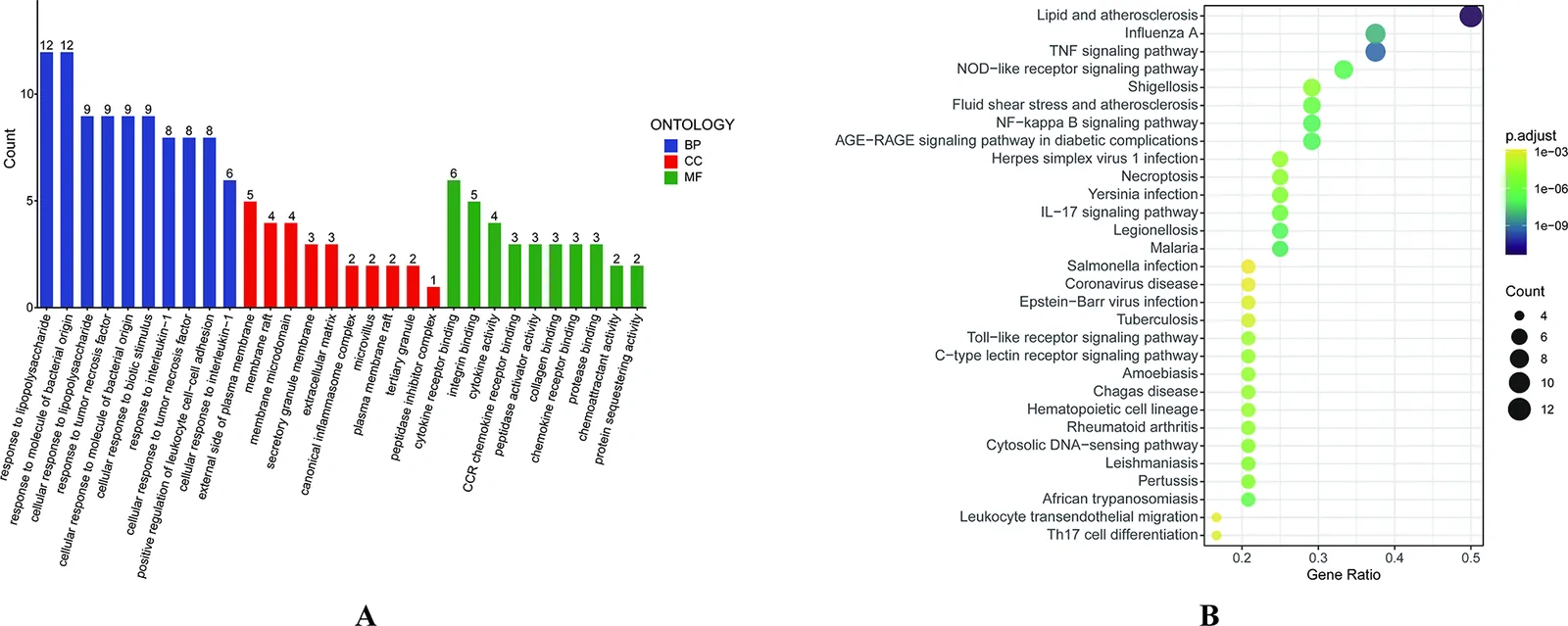
gene ontology (GO) functional annotation and kyoto encyclopedia of genes and genomes (KEGG) pathway enrichment of Kae‑RF‑related genes. **A** GO enrichment histogram showing the distribution of intersecting genes across three categories: biological process (BP, blue), cellular component (CC, red), and molecular function (MF, green). The X‑axis shows the GO term names, and the Y‑axis shows the gene counts. **B** KEGG enrichment bubble plot displaying the enriched pathways. The X‑axis represents the gene ratio (the number of target genes annotated to a given pathway divided by the total gene count for that pathway), and the Y‑axis lists the pathway names. Bubble size corresponds to gene count, while the color scale reflects the adjusted *P*‑value using a purple-to-yellow gradient indicating higher significance of enrichment. Enrichment analysis was performed using the clusterProfiler package with the Benjamini‑Hochberg method for multiple testing correction

To explore the functional landscape of the 25 Kae‑RF‑related genes, we performed GO term annotation and KEGG pathway enrichment analyses (Fig. 4A–B). The GO results indicated that, regarding BP, the intersected genes were significantly enriched in inflammation-related categories, including responses to lipopolysaccharides and bacterial-derived molecules, as well as tumor necrosis factor and interleukin-1 (IL-1) responses. Additionally, these genes were associated with the positive regulation of leukocyte adhesion, pointing to their role in recruiting and adhering immune cells under inflammatory conditions. For CC, the intersecting genes were significantly enriched in the outer plasma membrane, membrane rafts, secretory granule membranes, and inflammatory vesicle complexes. This localization implies that the corresponding gene products primarily reside in membrane signaling platforms and structures associated with inflammatory responses. Regarding MF, the intersecting genes exhibited significant enrichment in cytokine receptor binding, integrin binding, chemokine receptor binding, and collagen binding. This suggests that these genes are integral to cytokine signaling, cell adhesion, and matrix remodeling. Furthermore, KEGG pathway analysis revealed that these genes were significantly enriched in inflammation- and fibrosis-related signaling pathways, including tumor necrosis factor (TNF), NOD-like receptors, NF-κB, IL-17, and advanced glycation end products-receptor for advanced glycation end products (AGE-RAGE) pathways, as well as pathways related to lipid metabolism, atherosclerosis, necroptosis, and chemokine signaling. The functional context of Kae-RF-related genes primarily encompasses critical pathways, including the inflammatory response, NF-κB-mediated transcriptional regulation, immune cell chemotaxis and adhesion, and necroptosis. This finding offers an initial functional overview of the multi-target pharmacological profile of Kae. However, it is important to emphasize that this result illustrates the general enrichment characteristics of the gene set rather than the specific functional mechanisms associated with individual genes.

### Core gene identification via machine learning

**Fig. 5:**
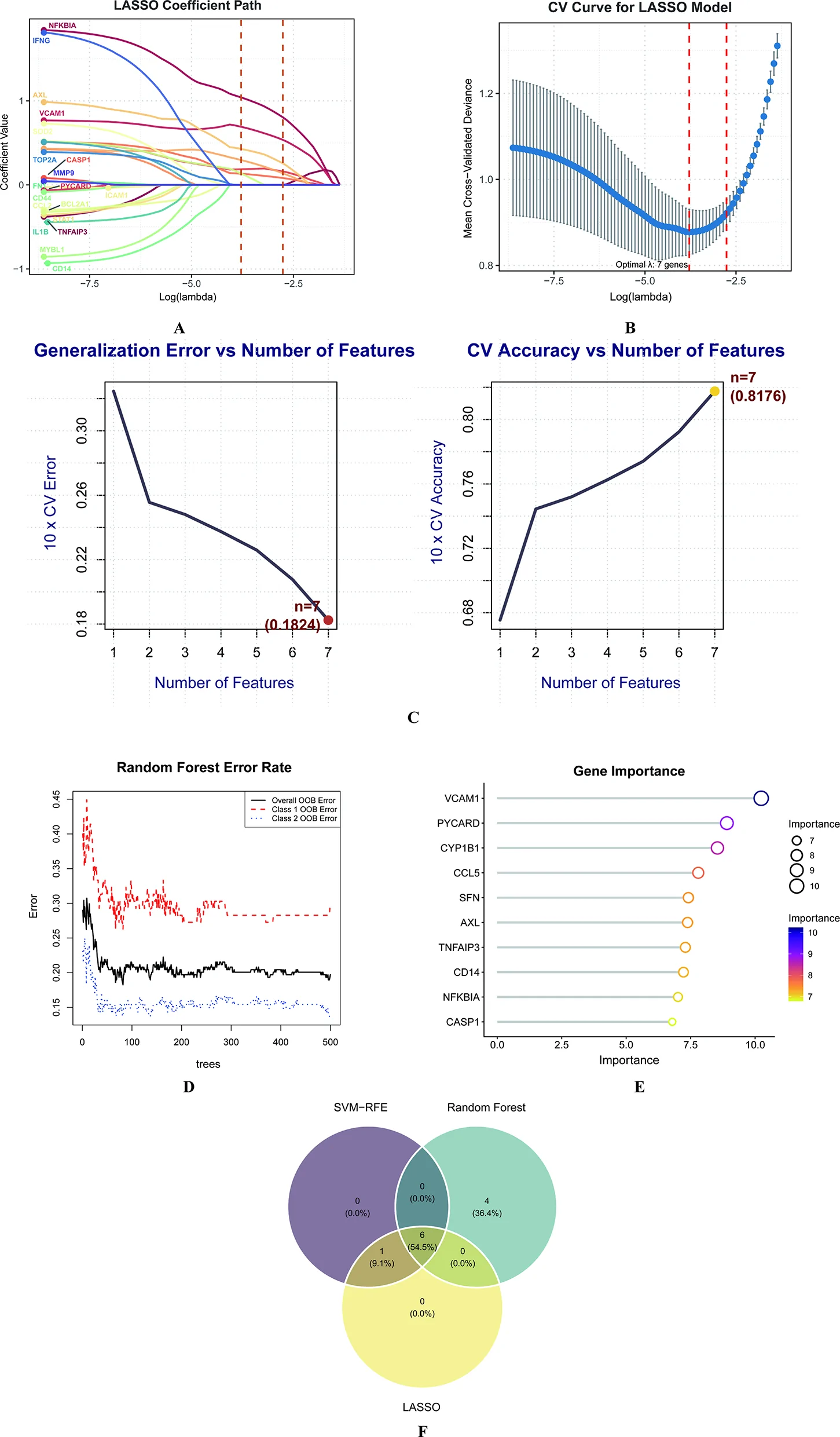
Identification of core Kae‑RF target genes by machine learning. **A** The least absolute shrinkage and selection operator (LASSO) regression coefficient path plot illustrates the trajectory of each gene’s coefficients as a function of log(λ), which gradually approaches zero with increasing λ. **B** The LASSO regression cross-validation curve displays the optimal λ value, indicated by the dashed line, selected through 10-fold cross-validation. The optimal λ corresponds to 7 genes with non‑zero coefficients. **C** Based on the support vector machine‑recursive feature elimination (SVM‑RFE) results, the optimal feature set comprised seven variables, which gave the lowest generalization error (0.1824) and the highest cross‑validation accuracy (0.8176). **D** The Random Forest error rate curve depicts the variation in error rate as the number of trees increases. The black solid line represents the overall out-of-bag error [Overall out-of-bag (OOB) Error], while the red dashed line and blue dotted line correspond to the out-of-bag errors for Class 1 (Class 1 OOB Error) and Class 2 (Class 2 OOB Error), respectively. **E** The Random Forest Gene Importance Plot illustrates the ranking of gene importance, with a color gradient from yellow to purple signifying increasing significance. Gene importance was assessed using the MeanDecreaseGini index. **F** The Venn diagram displays the intersection of the screening results from the three algorithms—LASSO, SVM-RFE, and Random Forest—resulting in a total of six core genes

To pinpoint the core genes through which Kae may act against RF, we applied three machine learning algorithms—LASSO regression, SVM-RFE, and Random Forest—for gene screening. Using the 25 Kae‑RF‑related genes as input, we performed LASSO regression with 10‑fold cross‑validation and identified 7 candidate genes with non‑zero coefficients (Fig. 5A–B). Subsequently, SVM-RFE conducted recursive feature elimination based on these 7 candidate genes, confirming the significance of all retained genes (Fig. 5C). Additionally, the Random Forest model identified 10 genes with the highest importance according to the MeanDecreaseGini metric (Fig. 5D–E). Finally, to obtain highly specific and robust core genes, we took the intersection of the candidates from the three algorithms using Venn diagrams, yielding a total of six core genes: VCAM1, NFKBIA, SFN, CCL5, CYP1B1, and AXL (Fig. 5F, Supplementary Material S2). This suggests that these genes may collectively form a key effector molecular network for Kae intervention in RF.

### Diagnostic performance assessment of core genes

**Fig. 6:**
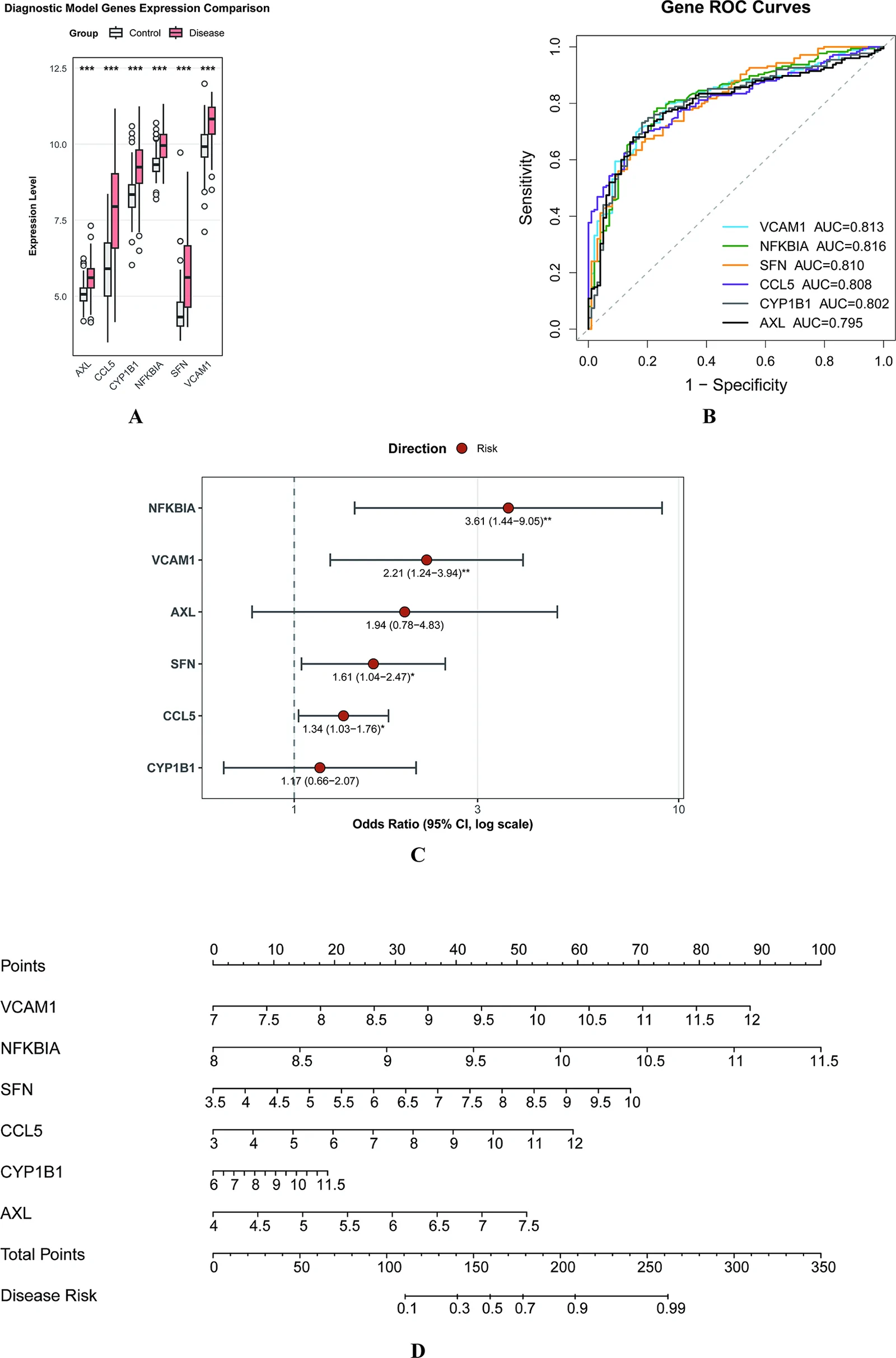

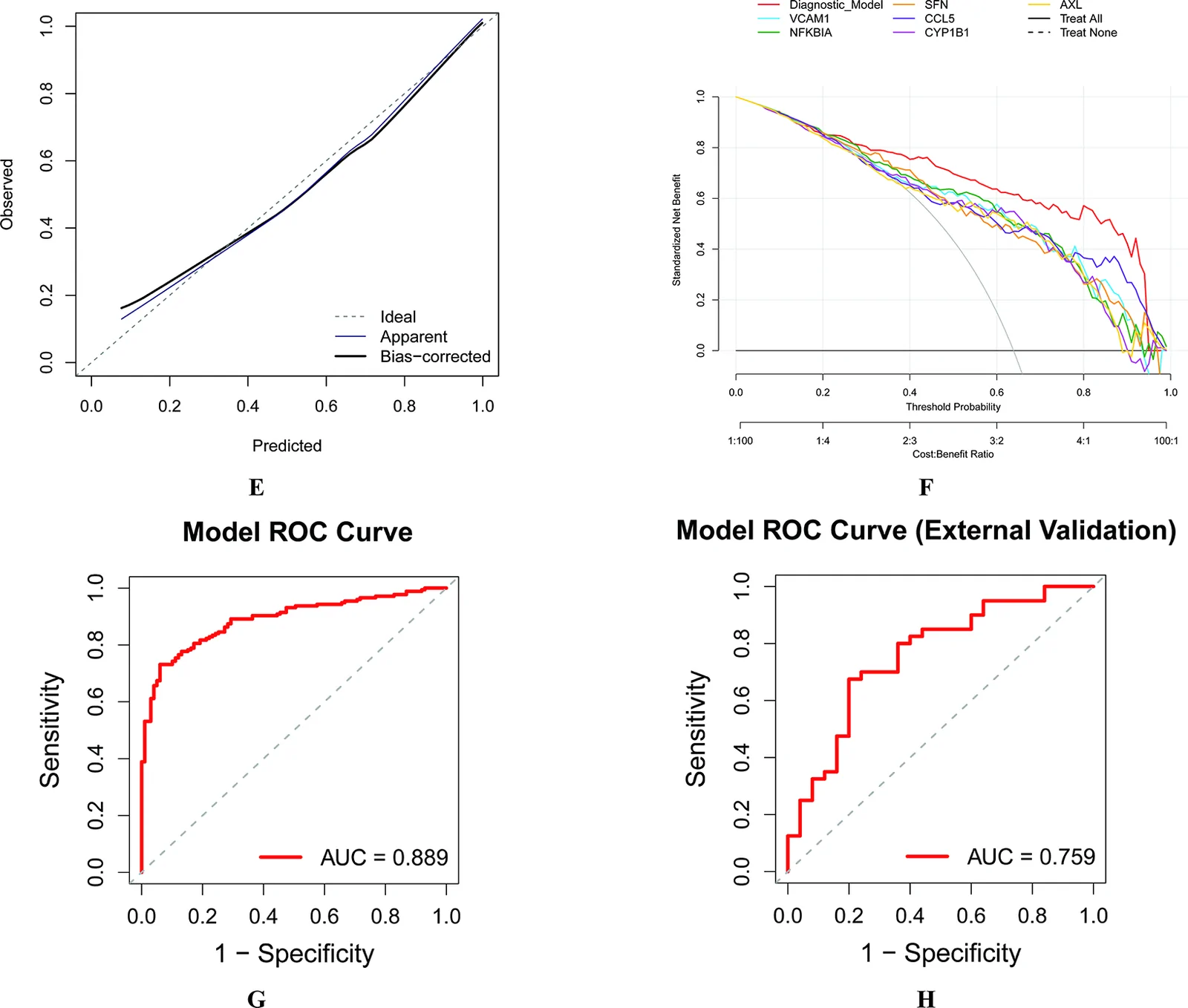
Diagnostic performance assessment and validation of multi-gene joint models.** A** Box-and-line plots illustrate the distribution of expression levels for the six core genes in the control group compared to the disease group. Box boundaries represent the interquartile range, and the internal horizontal line shows the median value. Whisker lines extending beyond the boxes denote the range of data distribution, while points outside this range are classified as outliers. Asterisks (***) signify *P* < 0.001. Statistical comparisons between groups were performed using the Wilcoxon rank‑sum test. **B** Single-gene receiver operating characteristic (ROC) curves depict the area under the curve (AUC) value for each gene, with the X-axis representing 1-specificity and the Y-axis representing sensitivity. **C** Forest plot showing the odds ratios (ORs) and 95% confidence intervals (CIs) derived from the multivariate logistic regression analysis. Red dots indicate OR estimates, and error bars represent 95% CIs; statistical significance is denoted by asterisks: *P < 0.05 and **P < 0.01. **D** Nomograms correspond to the probability of disease risk by mapping the expression values of each gene to their respective scores, accumulating total scores (ranging from 0 to 350) that correspond to disease risk probabilities (0.1–0.99). **E** Calibration curves illustrate the alignment of the model’s predicted probabilities with the actual observed probabilities. The ideal line represents perfect prediction, while the bias-corrected line reflects the model’s adjusted performance. **F** The decision curve analysis (DCA) plot illustrates the net clinical benefit across a range of threshold probabilities (X‑axis), with the standardized net benefit shown on the Y‑axis. **G-H** ROC curves show the AUC values of the combined multigene model across the training set and the external validation cohort (GSE22459). All ROC analyses were performed using the pROC package

To further evaluate the diagnostic value of the six core genes in RF, we generated box line plots using the whole‑transcriptome differential expression data. These plots illustrated the expression distribution of VCAM1, NFKBIA, SFN, CCL5, CYP1B1, and AXL in both the control and disease groups, revealing that all six genes showed significantly elevated expression in RF samples (Fig. 6A). Subsequently, we assessed the diagnostic performance of each core gene individually, with normalized expression levels serving as predictors. The ROC curves for the six core genes in the training set all yielded AUC values exceeding 0.70: VCAM1 0.813, NFKBIA 0.816, SFN 0.810, CCL5 0.808, CYP1B1 0.802, and AXL 0.795 (Fig. 6B), suggesting strong diagnostic efficacy for each gene. Building upon the single-gene diagnostic analysis, this study further developed a multigene joint diagnostic model. Multivariate logistic regression identified NFKBIA (OR=3.61, 95% CI 1.44–9.05, *P*<0.01), VCAM1 (OR=2.21, 95% CI 1.24–3.94, *P*<0.01), SFN (OR=1.61, 95% CI 1.04–2.47, *P*<0.05), and CCL5 (OR=1.34, 95% CI 1.03–1.76, *P*<0.05) as independent risk factors for RF (Fig. 6C). Based on the regression coefficients, nomograms were created to represent the probability of disease risk (0.1–0.99) by mapping the expression values of each gene to their corresponding scores and aggregating the total score (0–350). For instance, a higher expression value of VCAM1 correlates with a greater contribution score, resulting in a higher total score that reflects an increased probability of RF occurrence (Fig. 6D). The calibration curves indicate that the bias-corrected predicted probabilities align closely with the actual observed probabilities, indicating that the model is well calibrated within the training set (Fig. 6E). DCA revealed that the net benefit of the multigene combination model surpasses that of each individual gene, as well as the “Treat All” or “Treat None” strategies, over a wide spectrum of risk thresholds (0.2–0.9) (Fig. 6F). This finding suggests that the model offers substantial net clinical benefit. Furthermore, the overall AUC of the multigene model developed in this study was 0.889 for the training set and 0.759 for the independent external validation cohort (GSE22459), collectively indicating that the model exhibits strong diagnostic performance and generalization capability (Fig. 6G–H).

### SHAP interpretability analysis

**Fig. 7:**
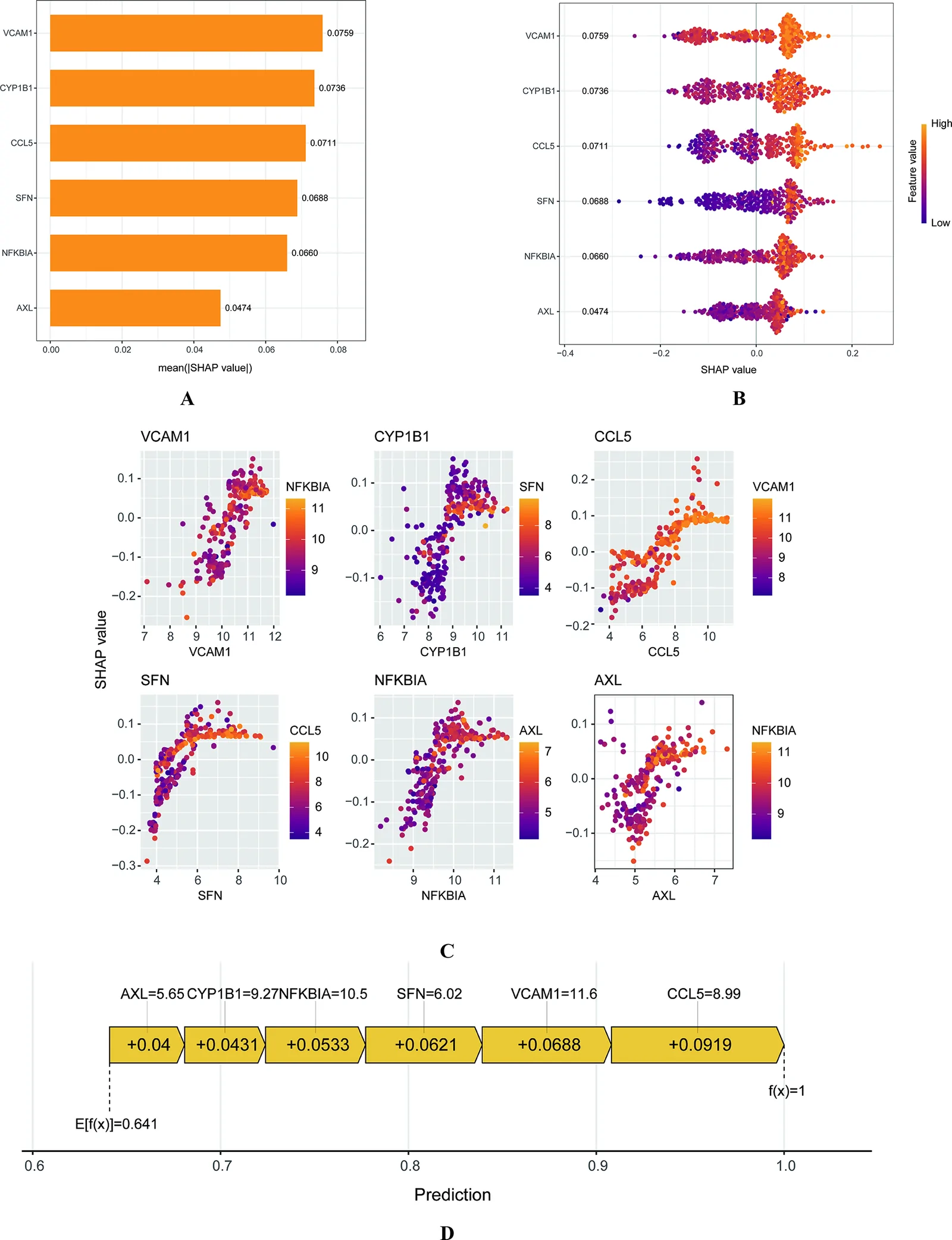
Shapley additive explanations (SHAP)-based interpretability analysis of core genes. **A** SHAP feature importance bar plot displaying the mean absolute SHAP values of the six core genes, ordered by contribution magnitude. SHAP values quantify each feature’s contribution to the model’s prediction output; higher absolute values indicate greater impact on the prediction. **B** SHAP summary plot showing per‑gene SHAP value distributions across all samples. The X‑axis represents SHAP values, and the Y‑axis lists gene names. Each dot corresponds to an individual sample, with dot color indicating expression level (purple: low; orange: high). **C** SHAP dependence scatter plot illustrating the non-linear association between a gene’s expression level (X-axis) and its SHAP value (Y-axis); the color scale denotes the expression level of the interacting gene (purple: low; orange: high). **D** The SHAP force diagram depicts the contribution of each feature to the prediction for a single sample. The baseline prediction E[f(x)] is adjusted by the SHAP value of each feature to derive the final prediction f(x), with features that positively contribute (increasing risk) represented in orange

To explore the internal workings of the multigene co‑diagnostic model and reveal the most influential features, we performed SHAP analysis for model interpretation. The ranking of SHAP feature importance indicated that VCAM1 (0.0759) and CYP1B1 (0.0736) exhibited the highest mean absolute SHAP values, followed by CCL5 (0.0711). In contrast, SFN (0.0688), NFKBIA (0.0660), and AXL (0.0474) displayed comparatively lower values (Fig. 7A), suggesting that VCAM1 and CYP1B1 are the most critical features predicted by the model. The SHAP summary plot (Fig. 7B) further revealed the association between each gene’s expression and its contribution to the model’s risk predictions. The results indicated that high expression levels of VCAM1, CYP1B1, and CCL5 contributed significantly to model predictions and were strongly associated with an elevated risk of RF, suggesting that these genes are primary drivers of the model’s predictions. Conversely, while high expression levels of NFKBIA, AXL, and SFN also positively influenced model predictions, their SHAP values were smaller, indicating a relatively weak association with RF risk. This suggests that these genes may play a secondary role in specific samples. This directionality aligns with the trend observed in the box-and-line plot, which showed that all six genes were significantly up-regulated in the disease group.The SHAP-dependent scatter plot illustrates the nonlinear effects of key features on model predictions. For instance, the SHAP values for VCAM1 and NFKBIA exhibited a stepwise increase with rising expression levels. Specifically, VCAM1 values surged from nearly 0 to above 0.15 when its expression surpassed 9.5, while NFKBIA displayed a comparable upward trend, indicating a significant expression threshold effect for both features. Additionally, a notable synergistic effect was observed between VCAM1 and CCL5; when VCAM1 was highly expressed (> 10) and CCL5 also exhibited high expression (> 8), the combined SHAP contribution of these two features exceeded 0.25, significantly surpassing the maximum contribution of either feature alone. Conversely, if the expression level of either feature was lower, the overall contribution diminished markedly, and the color gradient shifted from orange to purple. Similarly, the interaction between AXL and NFKBIA exhibited a positive stacking effect (Fig. 7C). These results suggest synergistic interactions among the core genes and their specific contributions to model decisions. SHAP was used to quantify each gene’s individual contribution to the baseline risk for individual sample predictions. The findings indicated that the positive bias was largely driven by VCAM1 (expression = 11.60, Δ = 0.069) and CCL5 (expression = 8.99, Δ = 0.092). Together with the positive contributions from other genes, these factors led to a model output of f(x) = 1, surpassing the baseline prediction of E[f(x)] = 0.641 (Fig. 7D).

### RF immune microenvironment and core gene-immune cell associations

**Fig. 8:**
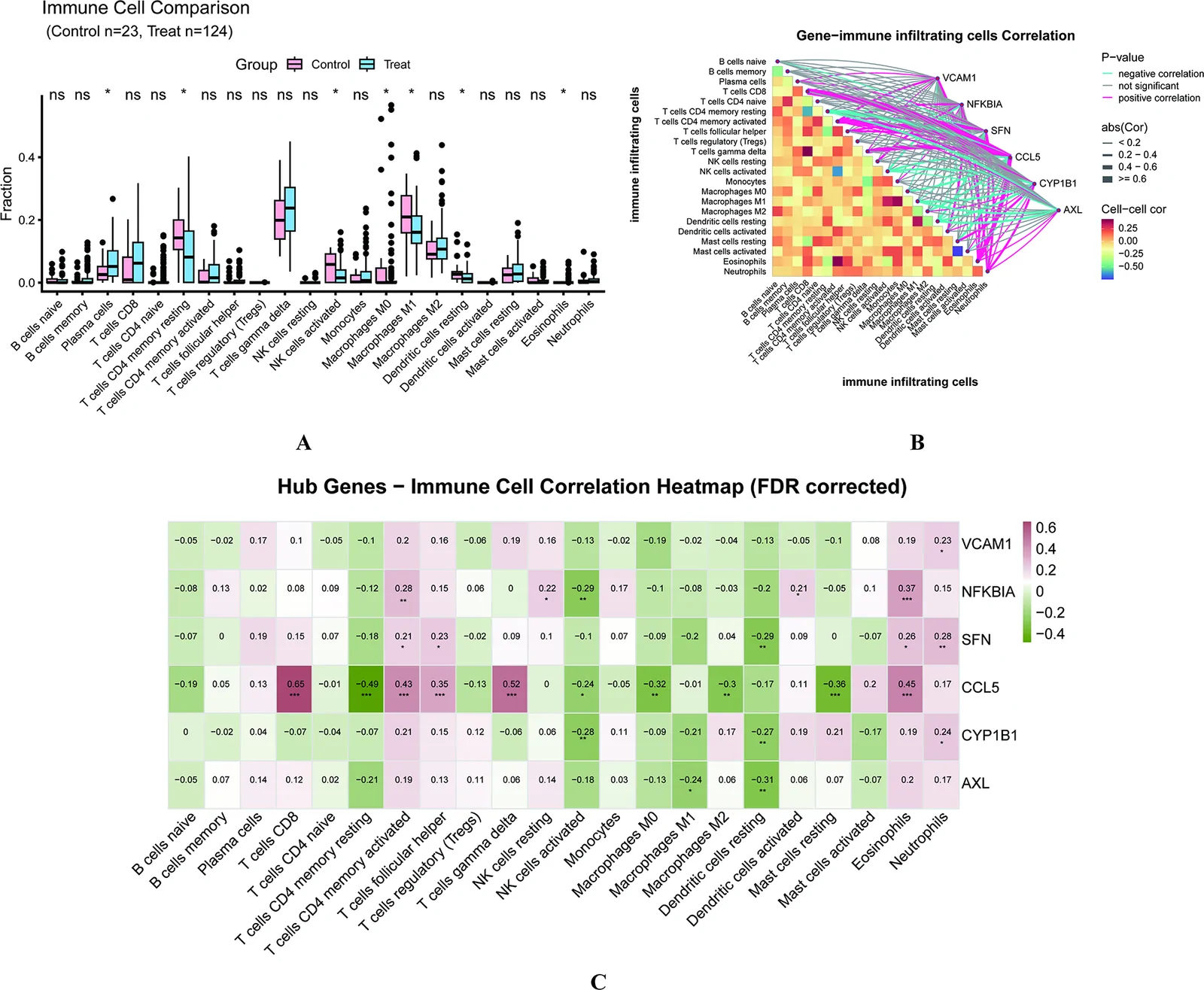
Immune infiltration environment and its correlation with core genes.** A** Box line plots illustrate the relative abundance of 22 immune cell subpopulations in control versus RF samples. The boxes represent the interquartile range (IQR), the horizontal line indicates the median, and whiskers denote the range of data distribution. **B** The association network plot depicts the correlation between core genes and immune cell types, with colors ranging from blue (indicating negative correlation) to red (indicating positive correlation) to represent the magnitude of the correlation. Line thickness reflects correlation magnitude, with thicker lines marking stronger significance; blue and pink indicate negative and positive correlations, respectively. **C** The correlation heatmap displays the Spearman correlation between various immune cell types and core genes. The correlation values are adjusted for false discovery rate (FDR), with colors ranging from green (indicating negative correlation) to pink (indicating positive correlation) to illustrate the magnitude of the correlation. Statistical analysis employed the Wilcoxon rank sum test for abundance comparisons and the Spearman test for correlation analysis. Significance levels are indicated by asterisks: *P < 0.05, **P < 0.01, and ***P < 0.001; ns denotes not significant. FDR correction was applied to all *P*‑values. All correlations with FDR‑corrected *P* < 0.05 were considered statistically significant

To assess immune cell infiltration in RF, we applied the CIBERSORT algorithm to estimate the relative proportions of 22 immune cell types across control and RF samples. Notably, the infiltration levels of seven immune cell types exhibited significant differences (Fig. 8A). Specifically, the proportions of plasma cells and eosinophils were markedly elevated in RF samples compared to controls (all FDR-corrected *P* < 0.05). Conversely, the infiltration levels of activated NK cells, resting memory CD4+ T cells, and M0 macrophages were significantly reduced in RF samples (Fig. 8A). Additionally, M1 macrophages and resting dendritic cells also demonstrated significantly lower infiltration proportions (FDR-corrected *P* < 0.05). Other immune cell subpopulations did not show statistically significant differences between the two groups, although this does not exclude their potential involvement in the disease process. Further investigation is needed to clarify their roles. Furthermore, Spearman correlation analysis (FDR-corrected) revealed correlations between six core genes and immune infiltrating cells (Fig. 8B-C). CCL5 showed positive correlations with eosinophils, CD8+ T cells, and γδ T cells, and negative correlations with resting memory CD4+ T cells, M0/M2 macrophages, and resting mast cells. NFKBIA displayed positive associations with eosinophils, activated dendritic cells, and resting NK cells, and negative associations with activated NK cells. SFN demonstrated a positive correlation with eosinophils, neutrophils, and activated CD4+ T cells, alongside a negative correlation with resting dendritic cells. CYP1B1 demonstrated positive association with neutrophils, while showing inverse association with both activated NK cells and resting dendritic cells. AXL was inversely correlated with resting dendritic cells and M1 macrophages. VCAM1 displayed a positive correlation with neutrophils (all FDR-corrected *P* < 0.05). Collectively, these results suggest that alterations in immune infiltration and core gene‑immune cell interactions may contribute to remodeling of the RF immune microenvironment and potentially link to disease progression.

### Quality control and immune profile identification

**Fig. 9:**
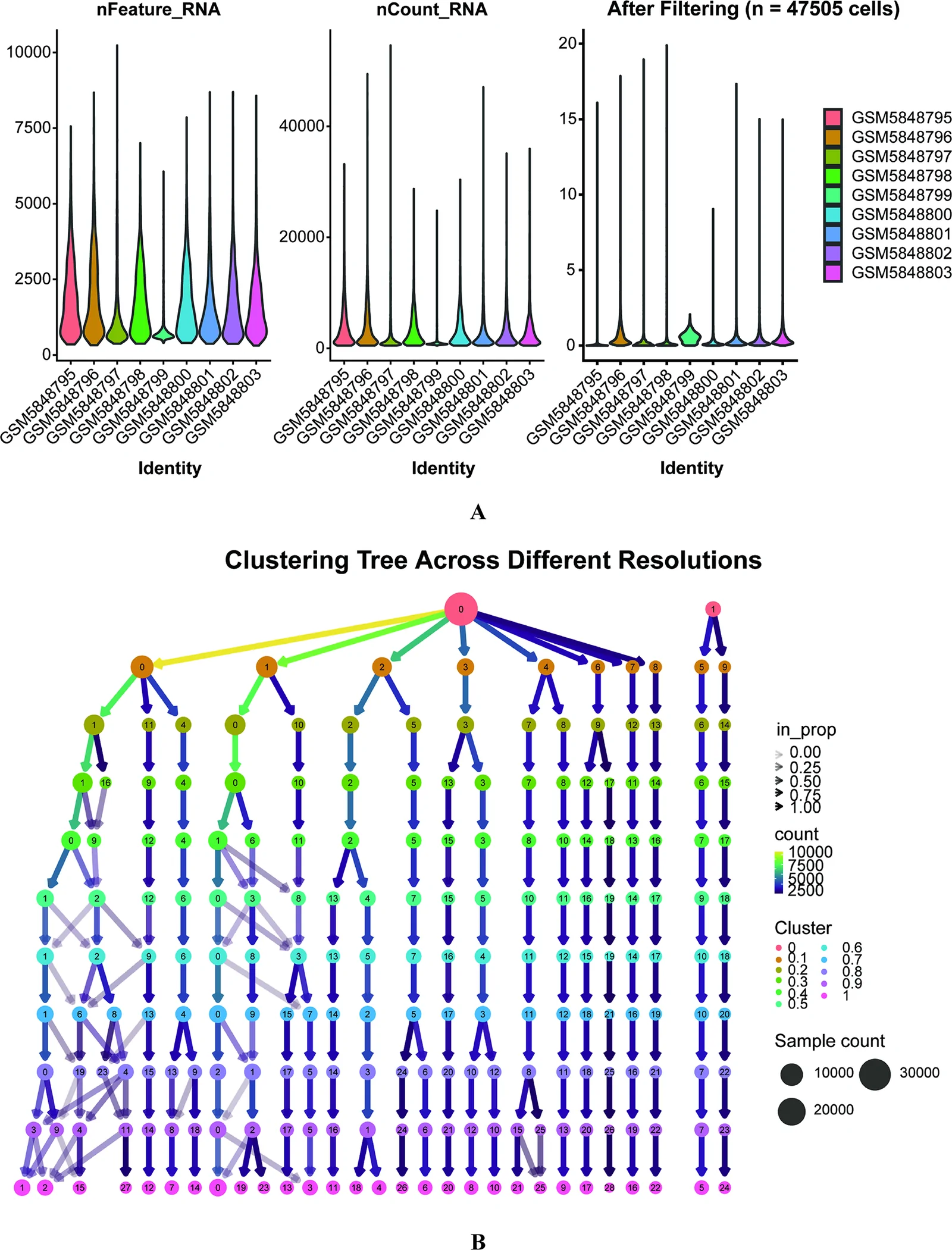

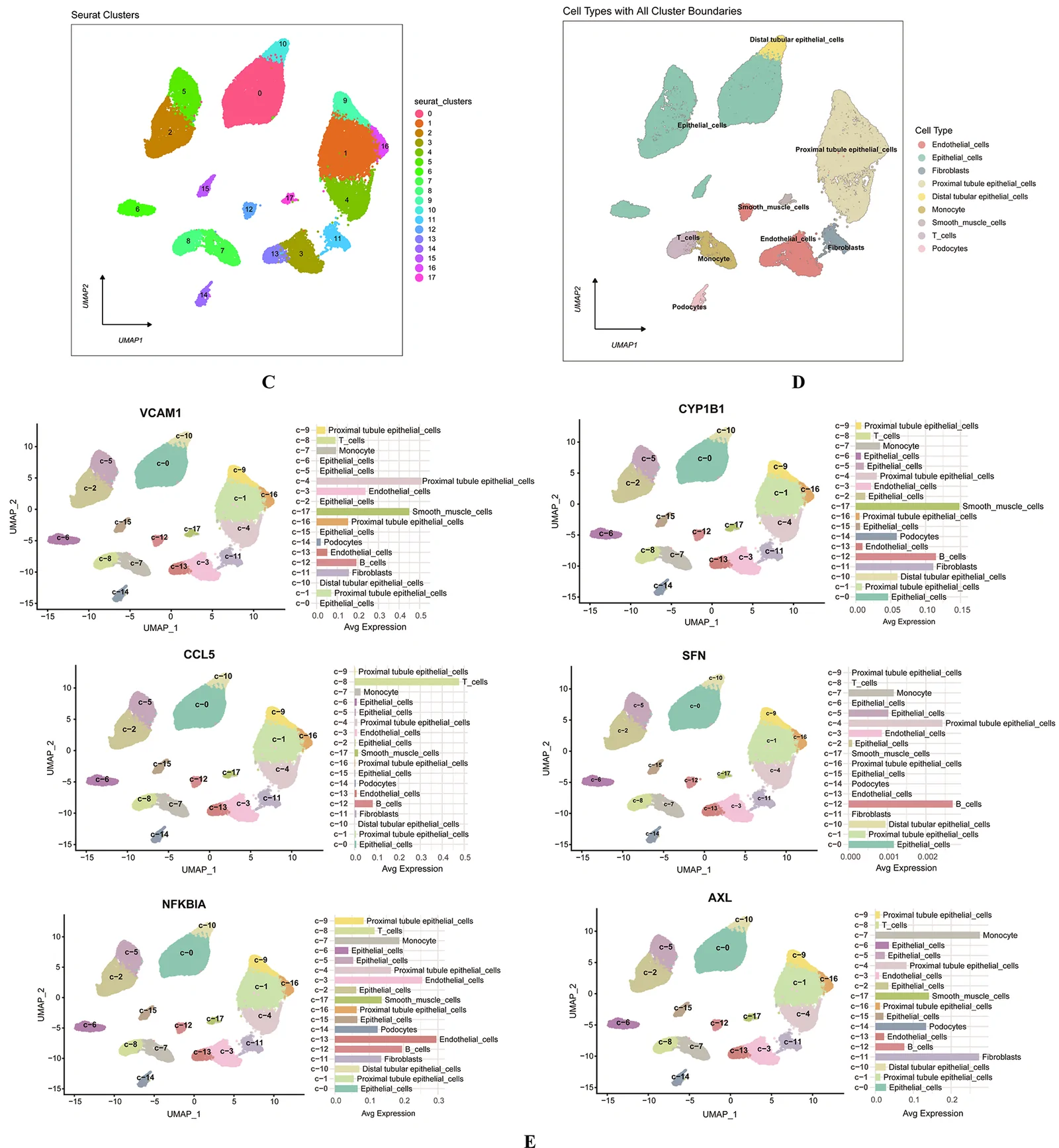

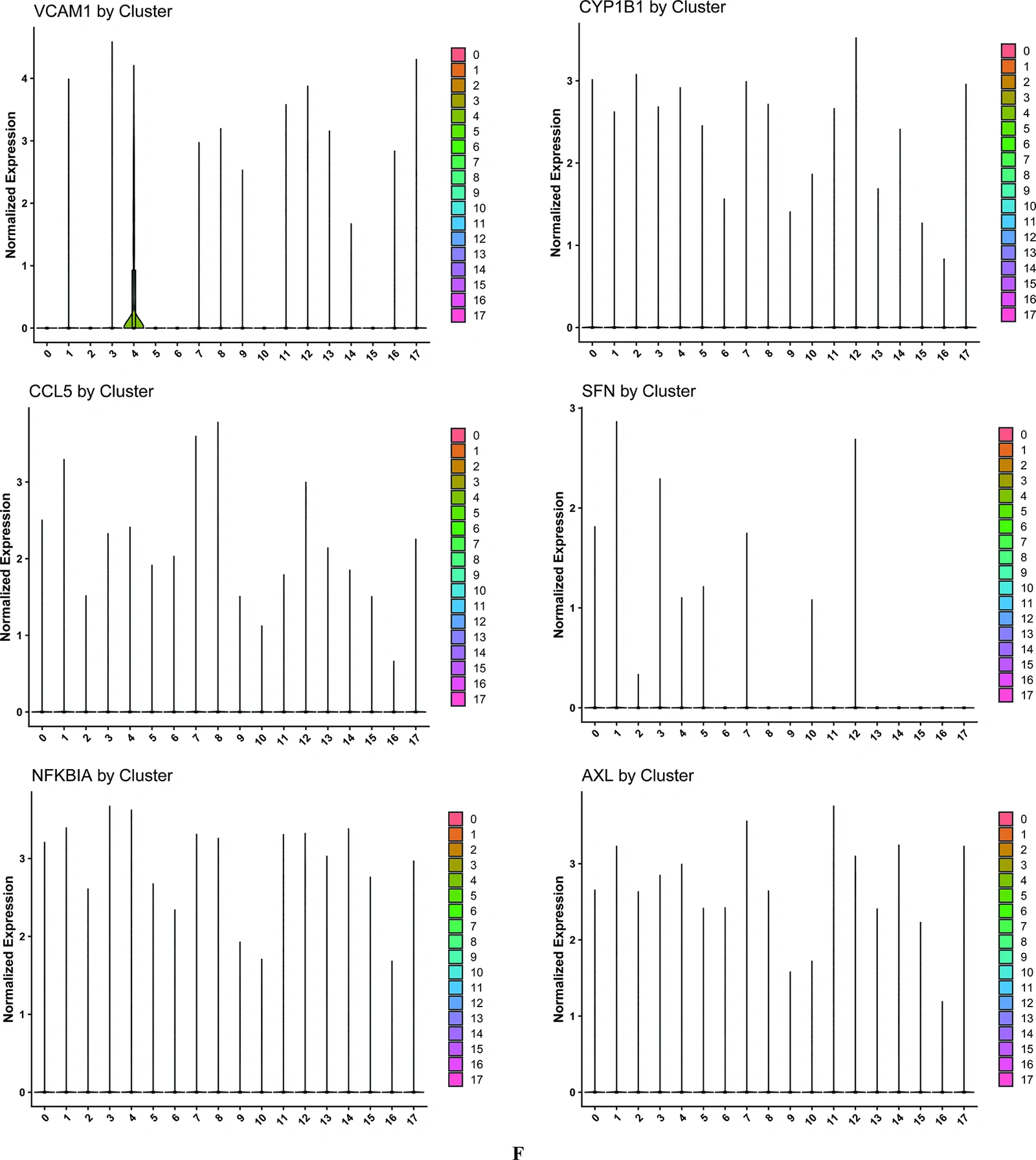

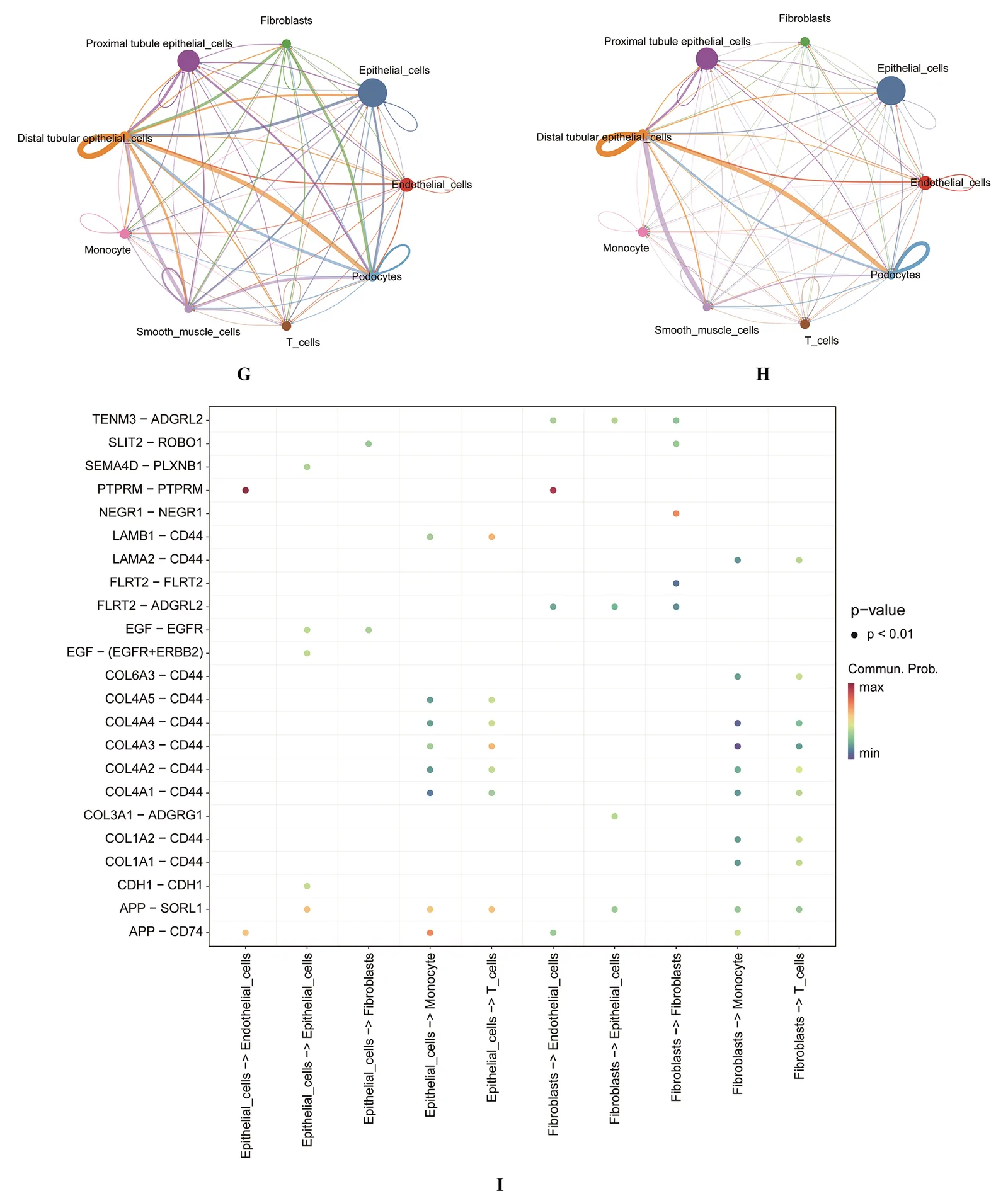
Single-cell immunoprofiling, core gene distribution, and intercellular communication in RF. Single-cell RNA sequencing data were derived from the GEO database (GSE195718), encompassing a total of 9 samples. **A** The violin plot illustrates the number of genes (nFeature_RNA), UMI counts (nCount_RNA), and the number of cells retained in each sample after filtering, resulting in a total of 47,505 cells. Cells with fewer than 300 genes or more than 20% mitochondrial genes were excluded. **B** The Clustree plot depicts the hierarchical relationships of clustering at various resolutions (0–1.0); node size reflects the number of cells within each cluster, while edge color indicates the in_prop value. **C** The uniform manifold approximation and projection (UMAP) visualization graph presents the cell clustering results identified by the Seurat algorithm, revealing a total of 18 distinct cell clusters. Each point on the graph represents an individual cell, with different colored regions denoting various cell clusters. Clustering was performed using the first 15 principal components (PCs) at a resolution of 0.3. **D** Another UMAP visualization graph displays the results of cell type annotation, encompassing nine primary cell types, each designated with a specific color. Cell annotation was performed using SingleR with reference to the Human Primary Cell Atlas. **E–F** Core gene localization plots illustrate the function and localization of core genes across different cell types within the single-cell dataset. The UMAP downscaled clustering plots feature distinct colored points representing various cell clusters, annotated with the names of the clusters and their corresponding colors. Violin plots depict the expression levels of each core gene across different cell clusters, with each cluster labeled by a unique number that corresponds to the UMAP plot. **G–H** The cell–cell interaction network graphs illustrate the connection counts and communication weights of interactions among various cell subpopulations. Node colors denote distinct cell types, while edge thickness reflects interaction strength; thicker lines indicate higher connection counts and greater communication weights. Intercellular communication analysis was performed using CellChat with the human ligand-receptor database (CellChatDB.human). **I** The intercellular communication bubble graph displays the probability and significance of cellular communication between different cell types. The horizontal axis denotes interacting cell‑type pairs, while the vertical axis lists the signaling pathways and molecules involved. Bubble color signifies the magnitude of communication probability, transitioning from blue (low probability) to red (high probability), while bubble size represents the significance of the *p*-value

To characterize the cellular heterogeneity of RF and the cellular origins of core genes at the single-cell level, we conducted a single-cell transcriptome analysis on nine samples from the GSE195718 dataset. Initially, we obtained a total of 47,505 cells following rigorous quality control, which involved filtering out cells with fewer than 300 genes and those exhibiting more than 20% mitochondrial gene expression (Fig. 9A). We then eliminated further environmental RNA contamination using decontX and filtered out cells with a contamination ratio exceeding 50%. Subsequently, scDblFinder was employed to remove doublets, resulting in 37,020 high-quality cells for further analysis. Clustree plots illustrated the stability of clustering results across various resolutions, indicating that a resolution of 0.3 effectively balances clustering granularity with biological relevance (Fig. 9B). The Seurat algorithm was then applied for clustering based on the first 15 PCs at a resolution of 0.3, leading to the identification of 18 stable cell subpopulations (Fig. 9C). Finally, automatic annotation using SingleR, referencing the Human Primary Cell Atlas and supplemented by manual corrections, identified cell types including endothelial cells, epithelial cells, fibroblasts, proximal tubule epithelial cells, distal tubular epithelial cells, monocytes, smooth muscle cells, T cells, and podocytes (Fig. 9D). Notably, a high proportion of epithelial cells and proximal tubular epithelial cells were observed.

### Cell type-specific distribution of core genes

The localization of core genes (Fig. 9E–F) exhibited distinct lineage specificity. VCAM1 showed the highest expression in smooth muscle cells, followed by endothelial cells and fibroblasts, while its expression in epithelial cells was minimal. This expression pattern correlated with vascular, mesenchymal, and myofibroblast lineages. CYP1B1 also showed the highest expression in smooth muscle cells, followed by fibroblasts and endothelial cells, indicating its potential role in myofibroblast activation. CCL5 was predominantly expressed in T cells and moderately in monocytes, reflecting a lymphoid-myeloid bipartite bias with T cells as the primary lineage. AXL was primarily expressed in monocytes and fibroblasts, exhibiting high levels in smooth muscle cells and relatively low levels in endothelial cells. Notably, smooth muscle cells and fibroblasts, which are the main sources of myofibroblasts in RF, displayed elevated expression of VCAM1, CYP1B1, and AXL, further underscoring the significance of these cell types in the fibrotic process. NFKBIA was relatively highly expressed in endothelial cells and monocytes, with a broader distribution. The expression of SFN across all cell types was extremely low (mean <0.005), suggesting potential silencing in steady-state renal tissues. These specific distributions provide a cytological foundation for understanding the potential functions of core genes within various cellular subpopulations in RF.

### Analysis of intercellular communication

In this study, the CellChat algorithm was employed to construct the intercellular communication network among various cell subpopulations within the RF microenvironment, identifying a total of 42 significant ligand-receptor interaction pathways (Fig. 9G–H). The communication network diagram revealed that distal tubule epithelial cells and podocytes served as the core nodes, while fibroblasts and epithelial cells were also extensively engaged in intercellular information exchange. Bubble maps further illustrated the significant ligand-receptor pairs that were highly enriched in each cell type (Fig. 9I). Notably, collagen family signals, such as COL1A1-CD44, were predominantly transmitted from fibroblasts to monocytes and T cells, indicating that fibroblasts may play a role in immune microenvironmental regulation via ECM molecules. Additionally, ECM-related communications, including COL4A1-A5 and COL6A3-CD44, were observed in interactions between epithelial cells and monocytes as well as T cells. Endothelial cells primarily functioned as signal receivers within the network (e.g., PTPRM-PTPRM, APP-SORL1). Monocytes and T cells, as immune subpopulations, occupy critical target positions in the efferent communication between fibroblasts and epithelial cells, suggesting their role as responders to ECM signals in the RF microenvironment. These findings provide insights into the intricate communication network of multicellular synergy in RF; however, the specific functional effects of each cell pair require validation through subsequent experiments.

### CYP1B1 expression dynamics and virtual knockdown during fibroblast activation

**Fig. 10:**
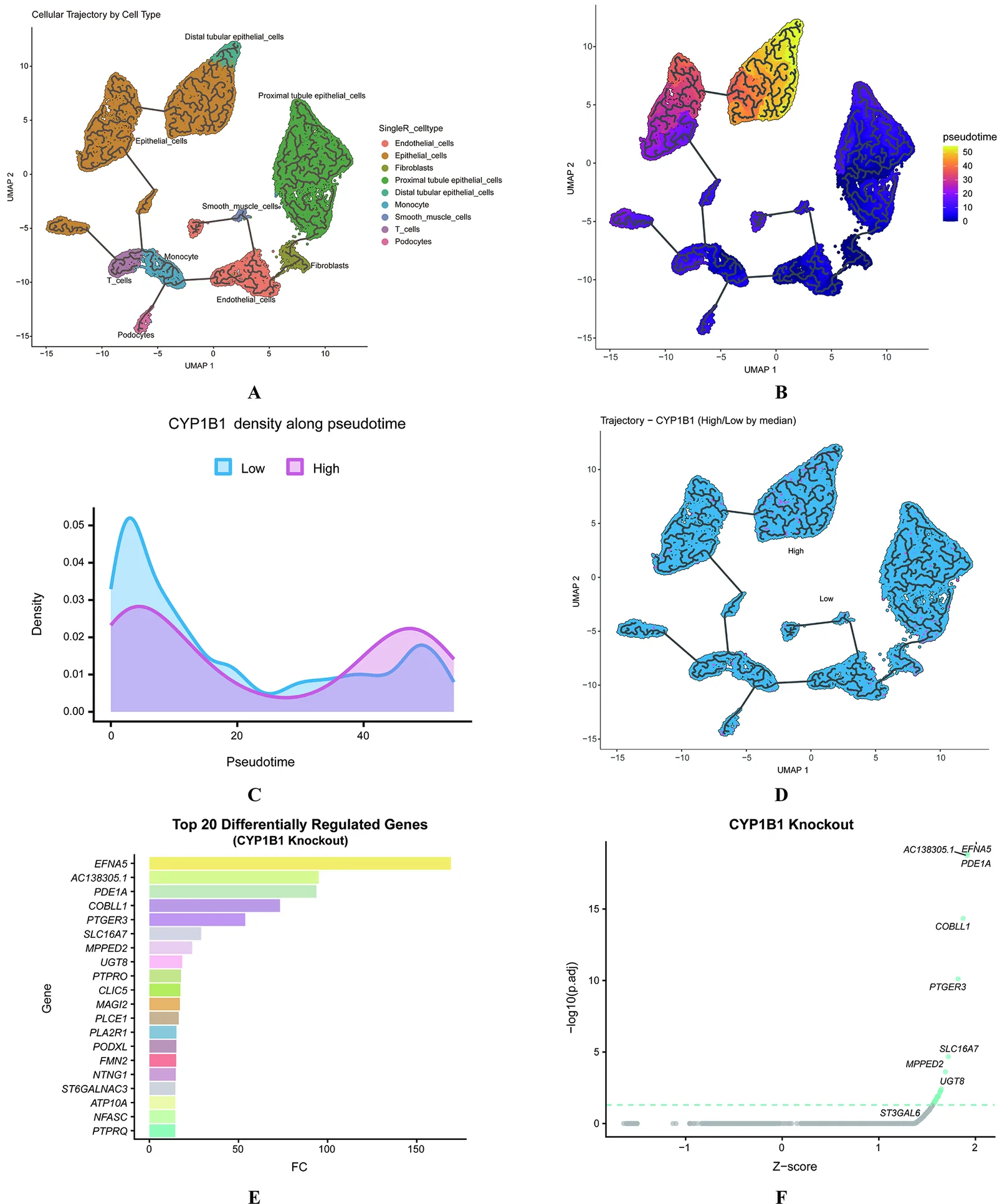
Fibroblast differentiation trajectory, CYP1B1 dynamic expression and virtual knockout simulation.** A** The UMAP visualization plot illustrates cell differentiation trajectories, with fibroblasts serving as the developmental starting point. Different colors represent cell types annotated by the SingleR algorithm, while grey lines indicate spindle trajectories and branching paths inferred by the Monocle3 algorithm. **B** The pseudotemporal analysis plot depicts the pseudotemporal distribution of cells in UMAP space, with color gradients transitioning from dark purple (low pseudotime, Pseudotime=0) to yellow (high pseudotime, Pseudotime=50). This gradient reflects the continuous dynamic process of cell evolution from an early state to a terminal state. **C** The CYP1B1 pseudotemporal density distribution plot illustrates the density curves of cells with high (pink) and low (blue) expression along the pseudotime. The X-axis represents pseudotime, while the Y-axis denotes density, highlighting the temporal enrichment characteristics of cell populations with varying CYP1B1 expression levels during the fibrotic process. **D** The CYP1B1 expression level trajectory map demonstrates the spatial distribution of cells with high (pink) versus low (blue) CYP1B1 expression, grouped by median along the differentiation trajectory, with each point on the map representing an individual cell. **E** The differential regulation gene histogram displays the 20 genes exhibiting the most significant differences following CYP1B1 virtual knockdown, arranged in descending order of Z-score. Virtual knockdown was simulated using the scTenifoldKnk algorithm. **F** The volcano plot resulting from CYP1B1 virtual knockdown shows all significantly dysregulated genes. The horizontal axis represents Z-scores, and the vertical axis represents -log10(*P*.adj). Green dots indicate significant differentially regulated genes with *P*.adj < 0.05

To trace the activation and transdifferentiation trajectories of fibroblasts during RF, we designated fibroblasts (cluster 11) as the developmental starting point and conducted a pseudotemporal analysis utilizing the Monocle3 algorithm. The findings indicated that the primary trajectory originated from fibroblasts and subsequently progressed through endothelial cells and smooth muscle cells, ultimately transitioning to epithelial cells and distal tubular epithelial cell states (Fig. 10A). Pseudotemporal analysis (Fig. 10B) revealed a gradual evolution of cells from the fibroblast initiation state (characterized by low pseudotemporal values, dark purple) to the terminal state (high pseudotemporal values, yellow-orange), thereby suggesting a wide spectrum of cellular phenotypic transitions throughout the RF process. Furthermore, proximal tubular epithelial cells, monocytes, T cells, and podocytes were also located in proximity to the trajectory, indicating the existence of intricate intercellular interactions within the fibrotic microenvironment. Dynamic analysis revealed that both CYP1B1 high- and low-expressing cells were initially distributed at the beginning of pseudotime, with a higher density observed in the low-expressing cells. As pseudotime progressed, the densities of both groups initially declined, followed by a gradual rebound, culminating in a pronounced peak during the late stage (Pseudotime≈40–50). At this point, the density of high-expressing cells significantly surpassed that of low-expressing cells (Fig. 10C), indicating that CYP1B1-overexpressing cells were notably enriched during the middle and late stages of fibrosis and may play a critical pathological role. The trajectory map (Fig. 10D) illustrated that CYP1B1 high-expressing cells were relatively densely concentrated in the late trajectory nodes, further supporting their involvement in the processes of cell activation or transdifferentiation during the terminal stage of fibrosis.

To further explore the functional significance of CYP1B1 within the RF transcriptional regulatory network, we employed the scTenifoldKnk algorithm to simulate a virtual knockdown of CYP1B1. The results indicated that the deletion of CYP1B1 resulted in the significant differential expression of 46 genes (*P*.adj < 0.05). The bar graph (Fig. 10E) illustrates the top 20 most differentially expressed genes, all of which were significantly up-regulated (FC > 10), with FC values exceeding 70-fold for genes such as EFNA5, AC138305.1, PDE1A, and COBLL1. This suggests that CYP1B1 may negatively regulate the expression of these genes. Scatter plots (Fig. 10F) revealed that the -log10 (*P*.adj) values for genes such as EFNA5, PDE1A, COBLL1, and PTGER3 surpassed the threshold line of *P*.adj = 0.05, thereby further confirming the statistical significance of the differences observed in these genes. The differentially expressed genes were functionally enriched in fibrosis‑related biological processes, including cell adhesion (EFNA5, COBLL1), G protein-coupled receptor (GPCR) signaling (PTGER3), ion transport (SLC16A7), and cytoskeletal remodeling (PDE1A) (Hu et al. 2023; Tsou et al. 2014; Wang et al. 2025; Wilczak et al. 2025). Coupled with the progressive upregulation of CYP1B1 expression along the activation trajectory of fibroblasts in the proposed temporal analysis, these findings are consistent with the hypothesis that CYP1B1 may serve as a functional target directly implicated in the pathological differentiation of fibroblasts into myofibroblasts, with its core regulatory network primarily oriented towards fibrotic effector processes such as ECM remodeling and cytoskeleton regulation.

### Molecular docking of Kae with proteins encoded by core genes

**Fig. 11:**
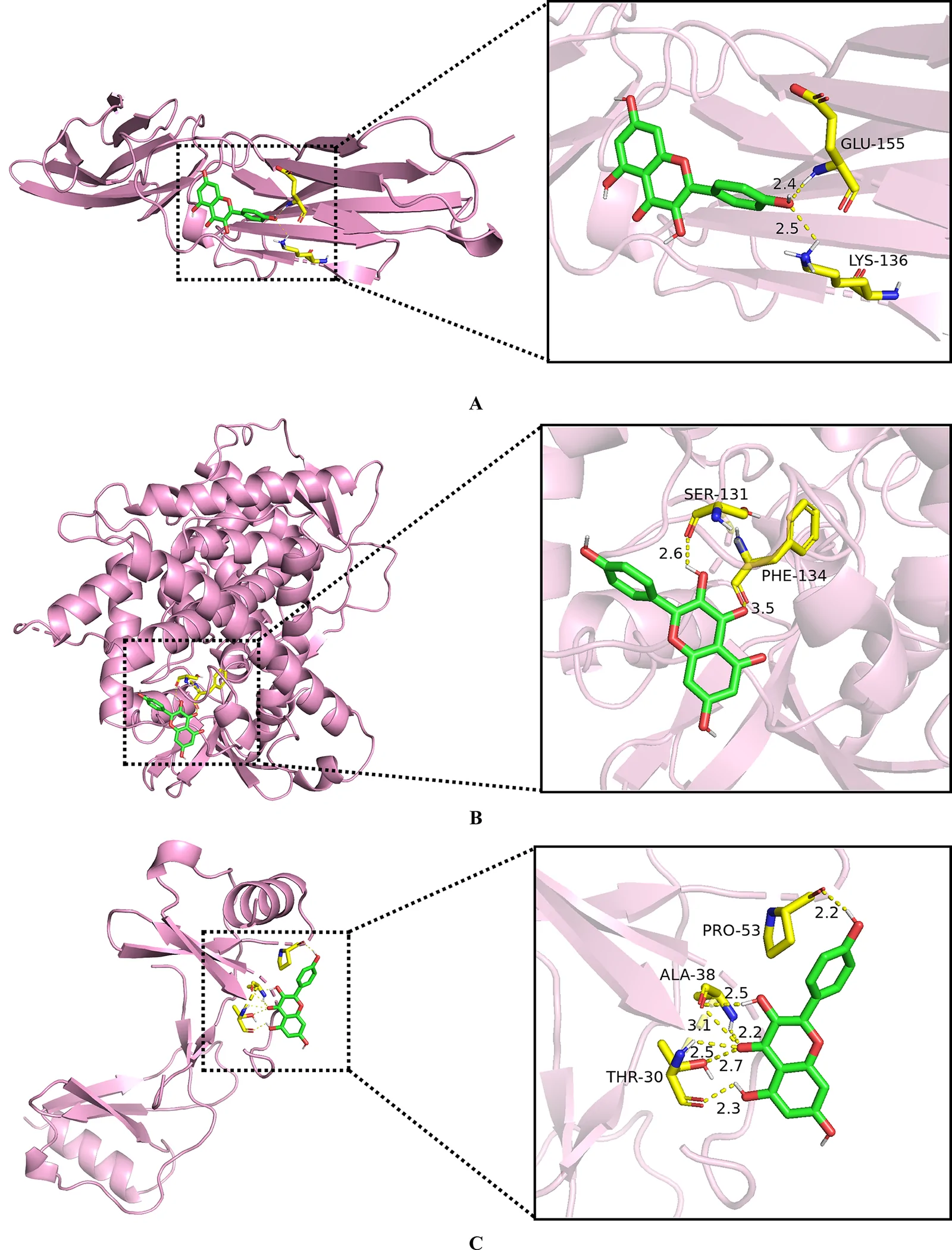

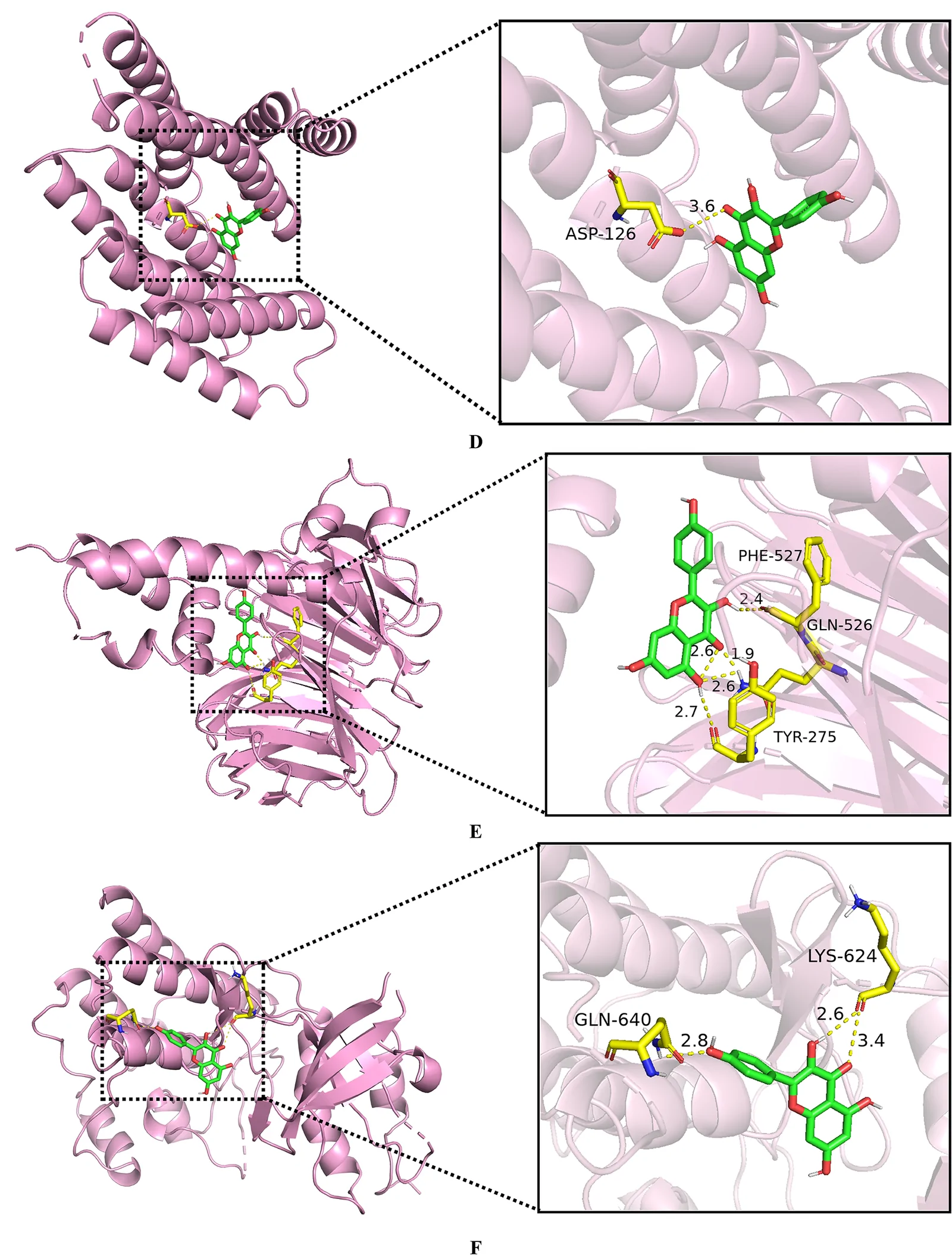
Molecular docking results. **A** Kae-VCAM1; **B** Kae-CYP1B1; **C** Kae-CCL5; **D** Kae-SFN; **E** Kae-NFKBIA; **F** Kae-AXL. Molecular docking diagrams showing the binding conformations of kaempferol with the core protein targets. The left panel displays the protein in a pink surface representation, offering an overall view of the complex. The right panel provides a detailed close‑up of the binding pocket, emphasizing the key interaction zones between the ligand and the protein. In this enlarged view, the ligand is depicted in green, while the amino acid residues are rendered in yellow and annotated with their standard designations. A dotted line between the ligand and the protein signifies the hydrogen bonding interaction, with the number adjacent to the dotted line representing the hydrogen bonding distance. Molecular docking was performed using AutoDock Vina. Binding energies for all six docking complexes are provided in Table 1; binding energies ≤ −5.0 kcal/mol were considered indicative of favorable binding potential

The molecular docking results indicated that Kae displayed a favorable binding potential for the proteins encoded by the six core genes (VCAM1, CYP1B1, CCL5, SFN, NFKBIA, and AXL), with all binding energies falling below −5.0 kcal/mol (Table 1). Visualization of the binding conformations (Fig. 11A–F) further revealed that all Kae-protein complexes formed docking conformations with significant binding potential, thereby offering a structural foundation for understanding their intermolecular interaction mechanisms.

**Table 1:** Molecular docking results: binding energies between kaempferol and the core target proteins

Ligand	Receptor	Binding energy (kcal/mol)
Kae	VCAM1	− 6.0
Kae	CYP1B1	− 6.5
Kae	CCL5	− 6.0
Kae	SFN	− 7.2
Kae	NFKBIA	− 7.0
Kae	AXL	− 5.3

## Discussion

The complex pathological process of RF is not solely driven by abnormal ECM deposition; rather, it results from a multilayered interaction involving chronic inflammatory progression, remodeling of the immune microenvironment, and abnormal activation of mesenchymal cells. This process is frequently accompanied by a synergistic dysfunction in the remodeling of the inflammatory signaling network and the activation of fibroblasts (Ding et al. 2026). Accumulating evidence has indicated that oxidative stress and chronic inflammation serve as key initiators and perpetuators of fibrotic progression, creating a vicious cycle of cellular injury, inflammatory cytokine release, and excessive ECM deposition (Wang et al. 2023a, b). Among the most extensively characterized pathways, TGF-β/Smad signaling plays a central role in promoting myofibroblast differentiation and ECM production, while Wnt/β-catenin signaling has been identified as a critical contributor to fibroblast activation and epithelial–mesenchymal transition (He et al. 2024; Miao et al. 2024). In addition to these canonical pathways, emerging evidence highlights the importance of metabolic disorders—including dysregulated lipid and mitochondrial metabolism—as well as gut microbiota dysbiosis in the pathogenesis of RF (Hong et al. 2025; Liu et al. 2025a, b, c; Zhao et al. 2025). These interconnected mechanisms collectively contribute to the initiation and progression of RF, underscoring the need for multi‑target therapeutic strategies. Kae, a naturally occurring flavonoid found in various medicinal plants and dietary sources, exhibits significant anti-inflammatory, antioxidant, antitumor, and antifibrotic properties (Liu et al. 2025a, b, c; Xu et al. 2026). However, systematic studies identifying its specific targets and molecular regulatory networks for improving RF are lacking. To address this gap, the present study integrated transcriptome differential analysis, WGCNA, multi-algorithm machine learning techniques (including LASSO, SVM-RFE, and Random Forest), and single-cell transcriptomics. This approach constructed a multi-dimensional analytical framework that spans from bulk transcriptome data to single-cell resolution. To further clarify the anti‑fibrotic mechanisms of Kae in RF, we carried out virtual knockout simulations and molecular docking analyses, thereby establishing a comprehensive computational research loop encompassing “key gene screening → cellular localization → pathological association → functional prioritization”. During the initial screening phase, functional enrichment analysis revealed that Kae-RF-related genes were predominantly associated with inflammation-related pathways, including NF-κB, TNF, and NOD-like receptors, aligning with the established anti-inflammatory properties of Kae. Nevertheless, these results represent the broader functional context of the gene set, and the specific functional roles of individual core targets remain to be experimentally investigated at the single-gene level.

Among the six core genes identified through machine learning, CYP1B1 was prioritized for subsequent functional investigation based on two criteria: (1) it exhibited the most pronounced temporal expression dynamics during fibroblast-to-myofibroblast differentiation in single-cell trajectory analysis, whereas the other five genes did not show comparable activation-associated upregulation; and (2) it occupies an upstream position in the transcriptional regulatory network, making it a plausible candidate for pharmacological modulation. Notably, VCAM1 showed the highest SHAP contribution but was not selected for functional analysis, as its dominant expression in endothelial cells suggested a primarily vascular rather than mesenchymal effector role.

To identify the upstream key molecules potentially regulated by Kae, we screened six core genes—VCAM1, CYP1B1, CCL5, SFN, NFKBIA, and AXL—from a total of 25 Kae-RF-related genes using multiple machine learning algorithms, complemented by SHAP interpretability analysis. Their viability as predictors for RF diagnosis was further supported by a multigene co-diagnostic model and independent external validation. Notably, VCAM1 (0.0759) and CYP1B1 (0.0736) exhibited the highest SHAP contributions, suggesting their potential role as the most representative predictors within the model. Importantly, although CYP1B1 did not yield the highest contribution to the diagnostic model, it occupied a pivotal upstream position in the transcriptional regulatory network. Following the virtual knockdown of CYP1B1, 46 genes were significantly upregulated, and its expression was progressively elevated throughout the fibroblast activation process. Existing studies corroborate that CYP1B1 expression is markedly upregulated in mouse models of hepatic fibrosis, and that the inhibition or knockdown of CYP1B1 effectively mitigates hepatic stellate cell activation and the progression of hepatic fibrosis (Tung et al. 2024). In the context of RF, Cao et al. reported that CYP1B1 mRNA expression is progressively increased in the obstructed kidney of unilateral ureteral obstruction (UUO) mice, concomitant with the activation of AhR signaling, and these changes are positively correlated with the progression of tubulointerstitial fibrosis (Cao et al. 2022). Moreover, Liu et al. further reported a mechanistic link between gut microbiota dysbiosis, microbial-derived tryptophan metabolites, and the AhR–Wnt/β‑catenin signaling axis in RF, wherein CYP1B1 serves as a downstream effector of AhR activation (Liu et al. 2026a, b, c). Importantly, both studies identified natural compounds—erythro‑guaiacylglycerol‑β‑ferulic acid ether (GFA) from Semen plantaginis and polyporusterone A from Polyporus umbellatus—that can alleviate RF by modulating AhR signaling and, consequently, suppressing CYP1B1 expression. These observations support the plausibility that kaempferol may exert its anti‑fibrotic effects, at least in part, through modulation of the AhR–CYP1B1 axis, consistent with the predicted binding affinity between kaempferol and CYP1B1 (− 6.5 kcal/mol) observed in our molecular docking analysis. Furthermore, in a mouse model of RF, CYP1B1 has been shown to regulate oxidative stress and inflammation by mediating the CYP1B1/reactive oxygen species (ROS)/mitogen-activated protein kinase (MAPK) axis, thereby influencing the RF process (Zhang et al. 2025a, b, c). Collectively, these findings suggest a significant functional relationship between CYP1B1 and RF pathology. We hypothesized that CYP1B1 may play a direct role in regulating fibroblast activation and phenotypic remodeling, positioning it as a functional target of interest in the RF process. This hypothesis aligns with the findings from subsequent single-cell trajectory analyses and virtual knockout simulations. In contrast to traditional clinical indicators of renal impairment, such as blood creatinine and urea nitrogen levels, the CYP1B1-centered regulatory network identified in this study may correspond to key upstream regulatory nodes involved in fibroblast activation and ECM remodeling during RF. Literature-based functional annotation of differentially expressed genes following virtual knockdown revealed that the transcriptional network governed by CYP1B1 primarily participates in cell adhesion, cytoskeletal remodeling, and ECM-related biological processes. These findings further suggest that the core function of CYP1B1 is likely to directly mediate the pathological differentiation of fibroblasts into myofibroblasts, positioning it as a candidate functional target in the fibrotic process. Notably, although CYP1B1 did not achieve independent risk factor significance in multifactorial logistic regression, and its single‑gene AUC (0.802) was somewhat lower than those of the other core genes, we note that diagnostic performance and functional relevance address fundamentally different biological questions. Logistic regression and ROC analyses evaluate a gene’s ability to discriminate disease status based on population‑level expression differences; however, these statistical measures may not fully capture a gene’s mechanistic role in disease pathogenesis. In contrast, functional relevance can be more appropriately assessed through biological context‑specific evidence. CYP1B1 was prioritized as a functional target based on multiple lines of evidence that transcend conventional diagnostic statistics, including its progressive upregulation during fibroblast‑to‑myofibroblast differentiation in single‑cell pseudotime analysis, its upstream regulatory position in the transcriptional network, and the extensive transcriptional perturbations induced by its virtual knockdown, which affected 46 genes involved in cell adhesion, cytoskeletal remodeling, and ECM deposition. These findings suggest that CYP1B1’s functional significance may be context‑dependent—particularly stage‑specific during fibroblast activation—and therefore may not be fully captured by population‑level regression models that treat disease status as a binary outcome. Thus, while CYP1B1 may not serve as a strong independent diagnostic predictor, its biological relevance as a therapeutic target remains supported by multidimensional functional evidence, and its inclusion in the six‑gene diagnostic model was primarily as a complementary screening tool rather than as an independent diagnostic marker.

Utilizing the aforementioned analytical framework, this study systematically evaluated the immune microenvironmental characteristics associated with RF. The findings revealed an increase in plasma cells and eosinophils within RF samples, while M0/M1 macrophages and activated NK cells were diminished compared to the control group. This indicates a significant remodeling of the immune microenvironment in RF. RF is not attributable to abnormalities in a single cell type; rather, it represents a complex process of microenvironmental remodeling driven by the recruitment of immune cells, the release of inflammatory mediators, and the response of mesenchymal cells. Furthermore, single-cell localization analysis revealed that CYP1B1 is predominantly expressed in smooth muscle cells and fibroblasts, both of which serve as critical sources of myofibroblasts in RF (Montezano et al. 2022). The cellular localization features described above, along with the direct regulatory role of CYP1B1 in ECM remodeling-related transcriptional networks suggested by virtual knockdown, collectively suggest that CYP1B1 may contribute to the fibrotic process primarily through its effects on mesenchymal effector cells. Integrating these findings with the intercellular communication analysis, our CellChat results indicated that fibroblasts engage in intensive communication with monocytes and T cells primarily through ECM-related signals (e.g., COL1A1-CD44). This observation suggests that fibroblast-derived ECM molecules may actively recruit and modulate immune cell function, thereby linking stromal cell activation to immune microenvironment remodeling. Collectively, a comprehensive pathological network can be proposed: Kae may modulate CYP1B1 activity, which in turn regulates fibroblast activation and ECM production, and these stromal changes may further influence immune cell infiltration and function through ECM-mediated signaling. This integrated framework—Kae–CYP1B1–stromal cell activation–immune microenvironment remodeling—illustrates the multi-layered nature of RF pathogenesis and suggests that the therapeutic effects of Kae may involve both cell-autonomous and microenvironment-mediated mechanisms. Additionally, Kae has been shown to mitigate multi-organ fibrosis by inhibiting NF-κB pathway activation and targeting TGF-β receptor I (Chen et al. 2025; Li et al. 2016), suggesting that its anti-fibrotic effects are characterized by synergistic actions across multiple pathways and targets. In light of the findings from the present study, we propose that CYP1B1 may represent a potential functional target for Kae in its anti-RF effects, with its core regulatory mechanism primarily involving potential regulation of fibroblast activation and ECM remodeling. Given its progressive upregulation during fibroblast-to-myofibroblast differentiation and its predominant localization in smooth muscle cells and fibroblasts, CYP1B1 may also hold potential as a biomarker for monitoring fibrotic progression, although this warrants further experimental validation. Taken together, this integrated framework—linking CYP1B1-expressing stromal cells to immune microenvironment remodeling through ECM-mediated communication—offers a potential mechanistic rationale for targeting CYP1B1 with kaempferol. It also suggests that the therapeutic effects of kaempferol in RF may operate through both direct modulation of fibroblast activation and indirect regulation of the immune microenvironment.

Pseudotemporal analysis traced the dynamic trajectory of fibroblast differentiation into myofibroblasts. The findings indicated that cells with high CYP1B1 expression were significantly enriched in the late pseudotime (~ 40 to 50), exhibiting a higher cell density compared to low-expressing cells. This observation suggests a temporal correlation between CYP1B1 and the maintenance of pathological status during the mid- to late stages of RF. Furthermore, it implies that CYP1B1 may be more closely associated with the sustained activation of fibroblasts, stabilization of the myofibroblast phenotype, and preservation of the pro-fibrotic microenvironment, rather than serving as an initiating factor in fibrogenesis. Concurrently, CellChat analysis revealed that fibroblasts engage in intensive communication with monocytes and T cells primarily through ECM-related signals (e.g., COL1A1-CD44). This interaction suggests that ECM signals between fibroblasts and immune cells may facilitate immune cell infiltration and inflammatory responses in fibrosis, thereby potentially forming a positive feedback network that accelerates fibrosis progression. Although CYP1B1 has been recognized as a key regulatory molecule in this process, its upregulation in fibroblasts during fibrosis progression, along with the extensive transcriptional perturbations induced by virtual knockdown, further supports its role as a pathologically relevant amplifying response molecule or a critical node with compensatory regulatory characteristics, rather than as a primary driver of fibrosis initiation. The cellular communication predictions presented in this study may be influenced by confounding factors, including cellular spatial proximity and the abundance of ligand/receptor expression. Therefore, further experimental validation of these conclusions is necessary. Based on our findings, we hypothesize that Kae may inhibit the pathological cascade of “fibroblast activation → ECM deposition” by disrupting fibroblast-mediated ECM signaling. Inflammatory factors and oxidative stress signals within the local microenvironment may induce a transition of fibroblasts from a resting to an activated state. The CYP1B1-overexpressing cells and the fibroblast-mediated ECM signaling communication axis identified in this study may serve as the primary effector units that respond to such stimuli and facilitate the progression of pathological fibrosis. This process, characterized predominantly by ECM overdeposition and the abnormal accumulation of myofibroblasts, constitutes a significant pathological basis for the ongoing deterioration of renal function as RF progresses to its advanced phases. Moreover, the expression of pro-fibrotic genes, including EFNA5 and PDE1A, was significantly up-regulated following the deletion of CYP1B1. This finding suggests that CYP1B1 may exert an anti-fibrotic effect by negatively regulating these genes. Additionally, it indicates that CYP1B1 could fulfill dual roles in stress response and homeostatic maintenance, depending on the disease stage or cellular context. Consequently, the elevated expression of CYP1B1 in the disease state likely represents an endogenous compensatory protective response. Insufficient up-regulation or impaired function of CYP1B1 may accelerate the progression of RF, while enhancing its function or sustaining its expression level could represent a novel strategy for RF intervention.

Additionally, this study employed virtual knockdown simulations utilizing scTenifoldKnk to further explore the hypothesis that CYP1B1 serves as a potential functional target of Kae from a computational biology perspective. As previously noted, the deletion of CYP1B1 induced extensive transcriptional perturbations, involving dysregulated genes functionally linked to fibrosis, such as those mediating cell adhesion, GPCR signaling, ion transport, and cytoskeletal remodeling (Hu et al. 2023; Tsou et al. 2014; Wang et al. 2025; Wilczak et al. 2025). This observation implies that CYP1B1 may play a role in the molecular network governing fibroblast activation and ECM deposition. It should be noted that these virtual knockdown results, together with the molecular docking findings, represent computational predictions that are intended to generate testable hypotheses and prioritize candidate targets for subsequent experimental validation, rather than to establish definitive causal relationships. The molecular docking results indicated that Kae exhibited favorable binding potential for CYP1B1 (binding energy − 6.5 kcal/mol), suggesting a predicted physical interaction; however, molecular docking alone does not predict whether Kae acts as an agonist or antagonist of CYP1B1 enzymatic activity. Importantly, our virtual knockdown data revealed that CYP1B1 loss-of-function led to upregulation of pro-fibrotic genes, which may appear to contradict the hypothesis that Kae improves RF via CYP1B1 inhibition. We interpret this apparent discrepancy as potentially reflecting stage-dependent dual roles of CYP1B1—pro-fibrotic in early phases and compensatory/protective in late phases—as supported by the pseudotime enrichment of CYP1B1-high cells at late differentiation stages. Therefore, rather than simply inhibiting CYP1B1, Kae may modulate its activity in a context-dependent manner—potentially supporting its protective role during late-stage fibrosis while counteracting its pro-fibrotic effects in early stages—a hypothesis that requires direct experimental testing. It is important to acknowledge that the molecular docking findings merely indicate potential binding tendencies and do not constitute direct evidence of pharmacological effects, which require validation through subsequent experiments. The virtual knockdown of CYP1B1 led to a significant upregulation of several known pro-fibrotic genes, such as EFNA5 and PDE1A (FC > 70), alongside other genes associated with fibrosis-related biological processes. Among these, some may represent protective responses, further underscoring the complexity of CYP1B1-mediated transcriptional regulation. Notably, the transcriptional landscape following CYP1B1 loss-of-function was not confined to a single direction of fibrosis regulation, but rather reflected a broad perturbation of multiple pathways. This broad transcriptional response indicates that CYP1B1 may inhibit the excessive activation of a diverse transcriptional program by negatively regulating these genes. Prior studies have indicated that the inhibition of CYP1B1 expression effectively mitigates renal and hepatic fibrosis, suggesting a pro-fibrotic role in these animal models (Tung et al. 2024; Zhang et al. 2025a, b, c). This finding may appear contradictory to the observed upregulation of several pro-fibrotic genes following CYP1B1 knockdown in the present study. However, when considered alongside the enrichment of CYP1B1 at late fibroblast activation stages in pseudotime analysis, we hypothesize that this discrepancy may arise from differences in disease stage. Specifically, CYP1B1 may exert pro-fibrotic effects during early-stage fibrosis while limiting excessive tissue damage in the late stage through compensatory upregulation—a stage-dependent pattern that raises the possibility of a protective role in later-stage fibrosis, warranting further investigation. Thus, we propose that the role of CYP1B1 in RF is not merely pro-fibrotic or inhibitory; rather, it encompasses a combination of contributions to pathological responses, regulation of oxidative stress, and maintenance of tissue homeostasis. Similarly, the role of Kae in ameliorating RF does not rely solely on a single target, CYP1B1, but is achieved synergistically through the regulation of multiple pathways, including inflammation, oxidative stress, fibroblast activation, and ECM remodeling. In summary, the findings of this study suggest that CYP1B1 may serve as a potential regulatory node in the remodeling of the RF mesenchymal microenvironment. Furthermore, targeting CYP1B1-related pathways with Kae may represent a promising strategy to intervene in the aberrant activation of mesenchymal cells and to ameliorate the pathological microenvironment of RF.

In this study, we systematically investigated the potential regulatory role of CYP1B1 in RF through a comprehensive bioinformatics analysis. Initially, WGCNA combined with various machine learning approaches identified CYP1B1 as a core target gene associated with RF, indicating its likely pivotal role within the transcriptional regulatory network. Subsequently, immune infiltration analysis revealed distinct microenvironmental remodeling features in RF tissues, characterized by increased infiltration of plasma cells and eosinophils, alongside decreased infiltration of M0/M1 macrophages and activated NK cells. Furthermore, single-cell transcriptomic analysis localized the predominant expression of CYP1B1 to smooth muscle cells and fibroblasts, suggesting its functional relevance to mesenchymal cell activation. Additionally, timing analysis and virtual knockdown simulations indicated that CYP1B1 may play a role in regulating the differentiation of fibroblasts into myofibroblasts, thus contributing significantly to mesenchymal remodeling in RF. Finally, molecular docking results indicated that Kae exhibits favorable binding potential with CYP1B1, providing preliminary evidence for considering CYP1B1 as a potential target for Kae’s therapeutic action. This analytical framework, spanning from screening to predictive validation, offers multidimensional insights into the biological significance of CYP1B1 in the context of RF. Although the six-gene diagnostic model was developed primarily for predictive purposes, the inclusion of CYP1B1 in this model—alongside its dynamic expression during fibroblast activation—provided the rationale for prioritizing it as a candidate functional target for Kae. We do not claim that diagnostic performance equates to therapeutic relevance; rather, the model served as a complementary screening tool to identify genes with potential biological significance. To further enhance the reliability of the findings, this study integrated bulk transcriptome and scRNA-seq data for cross-validation and comprehensive analysis. The bulk transcriptome analysis indicated that the diagnostic model based on the six core genes achieved an AUC of 0.889 in the training set and 0.759 in the independent external validation set (GSE22459), suggesting that the model possesses strong diagnostic predictive value and generalization performance. The scRNA-seq analysis revealed the CYP1B1-associated activation trajectory of fibroblasts at the single-cell level, along with its dynamic expression pattern. Collectively, the results of this study are consistent with the hypothesis that CYP1B1 may serve as a potential functional target of Kae to ameliorate RF, with its potential regulatory role likely involving modulation of fibroblast activation and extracellular matrix remodeling.

This work is subject to certain limitations. First, how CYP1B1 regulates fibroblast activation and ECM remodeling—as well as its potential indirect effects on inflammatory pathways such as NF-κB—is primarily derived from bioinformatics predictions and requires validation through in vitro and ex vivo functional experiments, including assessments of p65 nuclear translocation, IκBα (inhibitor of nuclear factor kappa-B alpha) phosphorylation levels, and other critical molecular evidence. Such validation should be extended to in vivo models such as UUO, complemented by co-culture systems involving primary renal fibroblasts, tubular epithelial cells, and immune cells to clarify cell-type-specific contributions to inflammation–mesenchymal crosstalk. To further validate these findings, CYP1B1 knockout or conditional knockout models should be employed to dissect the mechanism of action in RF, combined with assessments of transcriptional regulation, cellular function, and pathology. Overall, the multidimensional analysis presented in this study suggests that CYP1B1 may serve as a potential regulatory node closely associated with RF progression. Furthermore, it suggests that CYP1B1 may represent a potential target for Kae to exert anti-RF effects. However, whether it induces a pro-fibrotic effect, a protective compensatory effect, or both remains to be determined through in vitro and ex-vivo functional assays.

## Conclusion

Collectively, this multi‑omics and computational study prioritizes CYP1B1 as a potential functional target of kaempferol in RF. Integrative transcriptomic, machine learning, and single‑cell analytical approaches suggest that CYP1B1 may act as a context‑dependent regulator of fibroblast activation and ECM remodeling, with potential dual roles depending on the disease stage. Virtual knockout and molecular docking results provide preliminary evidence supporting its functional relevance and druggability. A six‑gene diagnostic model incorporating CYP1B1 shows encouraging predictive performance, thus warranting further clinical exploration. These findings offer a theoretical basis for future experimental validation and may guide the development of precision intervention strategies targeting CYP1B1 in RF.

## Supplementary Information

Additional file1 (XLSX 149 KB)

## Data Availability

The GSE datasets that support the findings of this study are available in the GEO database (https://www.ncbi.nlm.nih.gov/geo/, accessed on 19 March 2026) under the following accession identifiers: GSE76882, GSE22459 and GSE195718. The data are available upon request.
